# **Exploring the pattern of mental health support-seeking behaviour and related barriers among women experiencing intimate partner violence in urban slums of Bangladesh**: **perspectives from multiple level stakeholders**

**DOI:** 10.1371/journal.pgph.0004568

**Published:** 2025-05-09

**Authors:** Kamrun Nahar Koly, Jobaida Saba, Trisha Mallick, Fahmida Rashid, Juliet Watson, Barbara Barbosa Neves

**Affiliations:** 1 Health System & Population Studies Division, International Centre for Diarrhoeal Disease Research, Bangladesh (icddr, b), Mohakhali, Dhaka, Bangladesh,; 2 Social Equity Research Centre, School of Global Urban and Social Studies, RMIT University, Melbourne, Australia; 3 The Sydney Centre for Healthy Societies, School of Social and Political Sciences, University of Sydney, Australia; PLOS: Public Library of Science, UNITED STATES OF AMERICA

## Abstract

Intimate partner violence (IPV) is a recognised global public health concern, substantially impacting women's well-being. While there is growing research on how IPV victim-survivors seek mental health support in the Global North, it remains understudied in the Global South, particularly for those residing in slums in low-income countries like Bangladesh. Through interviews and group discussions with different stakeholders, this study explored the mental healthcare-seeking behaviour of victim-survivors of IPV residing in urban slums, barriers to service utilisation, and recommendations to strengthen care pathways. Stakeholders perceived IPV as normalised in slums, indicating sociocultural norms and interpersonal causes as significant contributors to mental health issues and events of IPV. Seeking healthcare and moral support for IPV from local dispensaries and informal sources was common; however, IPV victim-survivors had no knowledge about mental-health-related services. Low mental health literacy and lack of financial support prevented them from seeking the necessary care. Social stigma regarding accessing mental healthcare, coupled with the absence of professional service providers and community-based services, represent critical systemic challenges. Recommendations included promoting community-level awareness of IPV and mental health issues, increasing mental healthcare services, training health workers, and fostering positive masculinities in community-based interventions. Stakeholders emphasised the need to adopt culturally relevant interventions for tackling IPV and improving mental healthcare pathways, especially for the low-income population of Bangladesh.

## Introduction

Intimate partner violence (IPV) is a global public health concern and has been identified as a potential factor for women's adverse physical and mental health outcomes, such as depression, anxiety, PTSD, and suicidality [1,2]. The World Health Organization (WHO) declared IPV—which incorporates physical, sexual, and emotional abuse, as well as other controlling behaviours—as one of the most common forms of violence against women [3]. Worldwide, 27% of ever-partnered women aged 15–49 experienced IPV in their lifetime [4]. In societies where gender roles are unequal and families live in deprived areas with low socio-economic conditions, the prevalence of IPV against women is greater, varying from 55% to 95% [5–7]. IPV is common in developing country settings because prevailing gender norms place women in an inferior social position, limiting their decision-making power [8]. The role of gender norms might also contribute to explaining the prevalence of IPV in Bangladesh, but we lack evidence to support that connection. Further research is essential to clarify this connection and reveal the specific ways in which gender norms influence IPV trends in Bangladesh.

IPV has a multidimensional influence on women's health and well-being [1,9]. Evidence shows that most women who have experienced IPV are at risk of mental health conditions, including anxiety, depression, sleep problems, post-traumatic stress disorder (PTSD), and self-harm behaviour [10–12]. Predisposing factors, such as marginalised socio-economic status, low education, unemployment, and gender inequality, lead to the added burden of mental distress among women IPV victim-survivors [13,14]. Even though substantial evidence supports these bi-directional burdens, the care-seeking behaviour for mental healthcare among women who experience IPV is negligible [10,13].

Mental healthcare-seeking behaviour can be defined as actions considered for coping with mental health difficulties from external sources, which can be both formal (psychiatrists, psychologists) or informal (traditional healers, religious leaders, peers, family members, or other adults in the community) [15]. Most women (55–95%) from developing nations experiencing domestic abuse from their husbands opt for seeking informal over formal support [16–18]. In 2012, a Bangladeshi study reported that only 21% of women in the Dhaka slums disclosed spousal violence, and only 19% sought support from informal resources such as friends, relatives, and neighbours [18]. Similar trends were evident in a study reporting data across 31 nations: only 34.88% of IPV victim-survivors sought support, with the majority depending on family members [19]. In contrast, only 3.25% of women asked for assistance from formal institutions [19].

Likewise, a study in Zimbabwe found that victim-survivors of IPV from both urban and rural areas were less interested in seeking formal support due to believing they had to protect their marriages, the negative attitudes of service providers, privacy concerns, and a lack of resources [20]. Even in high-income countries like Australia, stigma, isolation, and partners' hostile behaviour impeded IPV disclosure, support seeking, and recovery [12]. Talking about or seeking support for IPV is often linked to shame and embarrassment or viewed as an issue not severe enough to report because of future fears, which in turn restrains women from seeking proper assistance, such as emotional and legal support [21,22]. Similarly, stigma and a lack of familial support significantly hinder individuals experiencing mental ill-health, serving as key barriers to accessing formal care—particularly for women [8,20,23]. Support seeking for IPV and the resulting mental distress is often less prioritised due to emotional factors, cultural and religious dimensions, and negative societal responses [21,24,25]. Frequently, women exposed to IPV are unable to leave abusive relationships because of financial dependency on their male partner [16]. This negatively impacts their decision-making ability, affecting help-seeking both for their experiences with abuse and with mental health difficulties [26]. Various other factors, such as individuals beliefs, practices, and inadequate mental health literacy, negatively influence women's mental health support-seeking behaviour, particularly of those residing in underprivileged communities like slums [26–28].

Urban slums are informal settlements with deteriorating, unsanitary structures, a degraded environment, and no access to basic amenities, including electricity, clean water, drainage systems, schools, medical facilities, and green spaces [29]. A majority (60%) of women from urban slums in Bangladesh experience abuse in their intimate relationships [28]. The burden of IPV is higher among slum dwellers than the non-slum population of Bangladesh [30]. Issues that affect slums, like extreme poverty, low quality of life, overcrowding, and lack of essential health amenities, were intensified during the COVID-19 pandemic and found to be associated with IPV [10,31–33]. Owing to the mobility restrictions imposed during the pandemic and fear of infection, mental health issues also increased in the Bangladeshi population in comparison to pre-pandemic periods [34,35]. Therefore, women residing in slums experience multiple adversities and vulnerabilities that can increase their risk of developing mental health conditions. However, there is a critical gap in our understanding of the mental health care-seeking behaviour of women who experience IPV in Bangladesh—and even fewer studies focus on those residing in slums. This is a pressing area to investigate, not only because of the detrimental consequences of IPV on vulnerable women's emotional, mental, physical, and social health, but also for economic reasons. Women are significant contributors to the national economy due to their role in both informal (household work, maid service, vendors, day labourers) and formal (garment industries) sectors [36]. To address these gaps, this study explored the mental health care-seeking behaviour of women who had experienced violence by an intimate partner in urban slums, as well as perceived barriers to accessing mental health services and multiple stakeholder recommendations to tackle the issue in Bangladesh. By identifying the factors hindering care-seeking behaviours of IPV victim-survivors in slums, we can provide urgent evidence to strengthen existing urban public health interventions targeting the overall health and well-being of people living in informal settlements such as slums. Findings from this study can further inform evidence-based safety net programmes for improving existing IPV-related support systems.

## Research design and methods

### Study design

This qualitative exploratory study involved stakeholder groups relevant to intimate partner violence and mental health sectors in Bangladesh. HealthCare researchers use this study design as it provides a deeper understanding of an issue's subjective and contextual nature, therefore allowing detailed exploration of related experiences and perceptions [37]. The findings are presented in a manner closely or directly resembling the research question from the participant's viewpoint. We therefore opted for this design to more thoroughly explore various stakeholders' diverse—and subjective—experiences and perceptions of IPV and mental health-seeking behaviours.

Data collection included different interview methods adjusted to stakeholders, such as in-depth interviews (IDIs), key informant interviews (KIIs), and focus group discussion (FGD). These were framed using semi-structured interview guides, drawing on several open and close-ended questions to help uncover information that might not be accessed in more structured and close-ended interviews [38–40]. Stakeholders were divided into seven groups (Table 1). We conducted face-to-face IDIs with slum community members (males and females), as this allowed better focus on the individual contexts of study participants while investigating complex and delicate matters related to their personal experiences of IPV and mental health seeking/perceptions [41]. Moreover, we aimed to gather their perspectives on the broader social and cultural elements inter-playing with IPV and mental health care-seeking behaviour. We also interviewed community leaders, healthcare providers, and gender specialists as key informants. KII was chosen for these participants, as it is designed to collect data from those with first-hand knowledge about a community or a research problem to understand the landscape of IPV and mental health care [42]. We conducted face-to-face KIIs with the community leaders and healthcare providers; however, we had to conduct online KIIs with gender specialists and an online FGD with mental healthcare service providers due to their busy schedules [43]. Their insights were expected to contribute individual and collective perspectives on the barriers and support mechanisms in mental healthcare provision in Bangladesh. A total of 59 participants contributed to the study, offering diverse perspectives on IPV and mental health within the community.

**Table 1:** Qualitative techniques and sample size.

Qualitative Method	Mode of data collection	Foci	Number of Participants
***In-depth Interview (IDI)***	Face-to-face	Women residing in slums	13
	Face-to-face	Men residing in slums	11
***Key informant interviews (KII)***	Face-to-face	Male community leaders	7
	Face-to-face	Female community leaders	7
	Online	Gender specialists	8
	Face-to-face	Health service providers	6
***Focus group discussion (FGD)***	Online	Mental health professionals (psychologists)	7
	**Total**		**59**

### Study settings

Bangladesh is a developing country with 63 million people living in urban areas. However, 52% of this urban population lives in slums, particularly in the capital city [44]. There are over 5,000 slums in Dhaka, where four million people live in single rooms, share water and sanitation facilities, and have poor access to household utility resources, health, and education [44]. For healthcare services, urban slum dwellers mostly depend on the local pharmacies and satellite clinics supported by NGOs (non-governmental organisations), and Health Service Delivery Projects are run by different organisations such as BRAC, Aalo Clinic, Simantik [45]. These healthcare services include distributing family planning materials, antenatal check-ups, delivery facilities, childcare, treatment, and medications for other regular health conditions at free or subsidised costs. NGOs such as Light House, BLAST (Bangladesh Legal Aid and Services Trust), and Ain o Salish Kendra (ASK) provide basic support to female residents of slums regarding violence against women. In terms of mental health care services, there are no community-based services available for people residing in slums. The National Mental Health Institute (NIMH) is the only specialised facility providing affordable mental healthcare in Dhaka. The mental health departments of the three public medical colleges based in Dhaka also offer cost-effective treatment options for mental health conditions [46].

To explore our research aims within slum settings, we conducted our study from June 1^st^ to December 31^st^, 2022, in five selected slums covered by the Urban Health and Demographic Surveillance Systems (UHDSS) of the International Centre for Diarrhoeal Disease Research, Bangladesh (icddr,b). These included Korail, Mirpur, Shampur, Dholpur, Tongi-Ershadnagar in Dhaka (North and South) and Gazipur City Corporation. The details of UHDSS are published elsewhere [47].

### Study population

The study used purposive and snowball sampling, as the combination of these techniques helps identify experienced or knowledgeable participants about the subject matter [48,49]. We identified potential participants from slum communities with the assistance of icddr,b-UHDSS team members based in the communities, known as Shastho Sebikas (health workers), who had been working there for more than seven years. Female community members with lived experience of IPV were drawn from a larger project on IPV, mental health, and household stress during the COVID-19 pandemic.

The long-term UHDSS staffs, known as icddr,b health workers, regularly visit the homes of a list of women registered within the UHDSS system. We utilised the PPS method (probability proportional to size) to identify participants for our study sample. All the slum areas were subdivided into multiple small slum zones based on their geographic locations, and the number of households was not uniform. After randomly selecting the zones of each of the five slum areas, we filtered the lists of women in those zones from the UHDSS sampling lists. Later, based on the size of the slum area and the number of eligible participants, we selected the women of the slum area using proportional intervals. For the umbrella project, we collected data from 405 women residing in slums, which was the required sample size with a 10% non-response rate. The inclusion criteria were: (a) aged 18–65 years; (b) married at the time of the survey; (c) lived with her husband for at least 1 year throughout the COVID-19 pandemic period; and (d) had lived in the slum for more than two years preceding the survey. We excluded women who were severely ill or pregnant, as their heightened vulnerability to prenatal depressive symptoms and hormonal fluctuations could potentially confound the study's findings. The health workers initially contacted the women from the participant lists by phone, and those currently in the slum zones who agreed to participate were invited to the survey for the umbrella project. The quantitative questionnaire included questions related to their demographics, COVID-19-related experiences, healthcare-seeking behaviours, and psychometric assessments.

Additionally, WHO standardised questionnaire on IPV was utilised to screen IPV victim survivors among respondents (Table 2), following previous studies conducted in similar low income settings [50,51]. The questionnaire collects data on the frequency of IPV, enabling assessment of its severity (none, moderate, severe) and type (physical, sexual, and/or emotional). Trained research team members administered the questionnaires; participants shared information verbally, and the researchers input that information in a written format. The data about IPV was only used for inclusion purposes and has not been analysed or presented in this study.

**Table 2:** Variables and labelling criteria of the WHO standardised questionnaire for identifying IPV.

Variables	Labels	Severity
Physical violence	Being slapped or having an object thrown at them Being pushed or shoved Being hit with a fist or object Being kicked, dragged, or beaten Being choked or burned intentionally Threatened with or having a weapon/knife used on them	Experiencing any of the six items was considered ‘physical violence.’ Moderate (experiencing only one or both of the first two items) Severe (having experienced any of the other four items)
Sexual violence	Physically forced to have sex Forced to have sex that made her afraid Presence of non-consensual degrading sex	Experiencing any of the three items was considered as *‘*sexual violence.’
Emotional violence	Being belittled or humiliated in front of others Subjected to fear or intimidation Either the participant or someone close to them being threatened	Experiencing any of the three items was considered ‘emotional violence.’ Moderate (if they only experienced one item) Severe (if they experienced two or more items)
Intimate partner violence	Experiencing any physical, sexual or emotional violence was considered as experiencing ‘intimate partner violence’	

For the qualitative data collection, we randomly approached 20 women from the umbrella project who experienced any form of violence according to the WHO questionnaire—five women from each of the four bigger slum zones covered by UHDSS. After gaining their consent, we also invited them to IDIs after finishing the quantitative data collection. Thirteen agreed to share their views in an interview; the remaining seven declined due to their busy schedules.

Before being invited to participate in the project, the women were informed about its objectives and assured of the privacy and confidentiality of the entire process, including data storage. Data collection was conducted at a time and place convenient for respondents to ensure their comfort and privacy, with efforts made to avoid the presence of others during the interviews. To confirm arrangements, respondents (females with lived experience of IPV) were contacted via phone to select their preferred venues for discussion. To ensure participants' safety and privacy, the interviews were scheduled during the day, when their husbands and in-laws were not at home due to most of the slum population being daily wage earners. Participants were also requested to refrain from sharing information about the data collection, interview process, or project with their husbands or others. We were able to conduct most of the IDIs (n = 9) with the women living in slums at their homes, but in some cases, interviews (n = 4) were conducted in shops or while walking along the roads to accommodate participants' circumstances. In instances where interviews were interrupted by guests or neighbours (n = 4/9), data collectors revisited participants to complete the process. The study data was collected in 2022 from slums where icddr,b's urban demographic surveillance system has been active since 2015. As a result, the community was familiar with the data collection process, and health workers accompanied data collectors during the interviews to ensure the process was conducted smoothly and securely. Through these measures, the safety and privacy of participants were strictly upheld.

In addition, we recruited married adult men aged 21–65 years (the age of marriage in Bangladesh is 21) who had been residing in the community for at least the five years preceding data collection. Men residing in slums included in this study were not identified as perpetrators; instead, we explored their general perceptions and attitudes about IPV, influential factors, and their impacts on mental health, since men play a pivotal role in changing societal norms [52]. We did not ask questions that might imply or suggest identifying individuals as perpetrators. To prevent any potential issues, we sought assistance from our UHDSS gatekeepers, who were familiar with the community and could identify the husbands of the participants selected for data collection.

Male and female community leaders (house owners or any person actively involved in slum administration or influential in the community) were chosen for this study because they were well-versed in their local area, actively involved in managing slum matters and resolving disputes, and well-accepted by local inhabitants. The UHDSS gatekeepers helped us identify the community leaders who had been living in the selected slums for over 15 years and were over 30 years of age.

We also identified healthcare providers who were pharmacy owners or worked in different healthcare related NGOs in the neighbourhoods through local UHDSS offices and via people residing in the slums. We interviewed healthcare providers in their pharmacies and office spaces. The insights of healthcare providers were gathered as they are considered the first point of contact regarding any physical or mental health-related issues inflicted by IPV [53].

Finally, we approached some of the professionals who work in the gender and mental health sectors of Bangladesh through the authors' established networks. Gender specialists were involved to bring their outlook on the gender-specific aspects of IPV and the mental health of slum residents. Mental healthcare service providers were included to discuss the barriers and opportunities of the mental health system to ensure care for IPV victim-survivors. The men residing in slums preferred meeting in their shops, tea stalls, or workplaces. The landlords preferred their own houses. Other leaders invited us to their community association offices. No identifiable information was collected from any participants during data collection. Instead, unique identity codes were assigned to each participant to ensure anonymity.

### Data collection

Recruitment for this study was completed from July 24^th^ until September 4^th,^ 2022. All stakeholders from the slums and healthcare providers were interviewed face to face by four research team members with master's degrees in medicine and public health and prior experience in qualitative data collection. The gender specialists and mental healthcare service providers joined online interviews and focus group conducted by the research team lead, a global mental health researcher. Interviewers and other research team members took notes during interviews and focus groups to record participants' expressions, notions, context, and notable moments, which later helped the coding process and triangulation sessions [54]. Moreover, we kept the notes to gather preliminary findings and double-check transcription accuracy.

We combined face-to-face and online data collection techniques. Face-to-face data collection allows for synchronised communication and facilitates understanding in-situ social cues [55]. On the other hand, online data collection is time- and cost-efficient, reducing geographical barriers, particularly during a pandemic [38,56]. Both methods are commonly employed in qualitative studies.

Building on relevant literature on IPV and mental health in similar LMIC settings [52,53,57–59], five separate semi-structured interview guides were developed for KIIs, IDIs, and the FGD. We initially tested these interview guidelines among residents, community leaders, and pharmacy owners in the slums of Mohammadpur and Malibagh, where icddr,b was running other urban projects. These slums were not part of UHDSS. After conducting six individual interviews among each group, we refined the guides by addressing challenges encountered during the interviews and incorporating feedback from the responses. The guides (S1 File) explored the current state and social perceptions of IPV, its relationship with mental health, the support-seeking behaviour of people residing in slums, and perceived recommendations for strengthening existing support services. Interviews took between 30–40 minutes, and the focus group took 165 minutes. All interviews and discussions were audio-recorded and transcribed verbatim by the research team. Key components were translated into English. All transcripts and translations were quality-checked by the co-investigators.

### Data analysis

Interview data were thematically analysed, using both inductive (identified in the data) and deductive codes (based on pre-existing ideas from our research aims) [60]. The research team familiarised themselves with the interviews by independently reading and coding the transcripts manually to define initial codes. The team then collectively agreed on a final code-book applied to the dataset. Codes were aggregated into sub-themes and themes, capturing all stakeholder data, and illustrative verbatim quotes were selected from the transcripts. Data triangulation was ensured by multiple meetings with the research team, during which the perspectives of the various stakeholders in involved in the study were considered. Participants were provided specific identification codes to ensure anonymity (S1 Table). We adhered to the consolidated reporting criteria for qualitative studies (COREQ) (S1 Checklist) [61].

### Ethical assurance for the protection of human rights

The study was carried out in accordance with the Institutional Research Ethics guidelines and ethical guidelines involving human participation (i.e., the Helsinki Declaration). The study was reviewed and approved by the Institutional Review Board (IRB) of icddr,b, which included a research review committee and ethics review committee (PR-22001). Detailed information about the study was described to participants before the interview. Before data collection, we obtained informed verbal consent and recorded the consenting process for male slum community members, gender specialists, and mental health professionals. Written informed consent was obtained from healthcare providers before their interviews took place. We did not obtain written consent from the women residing in slums; we only obtained informed verbal consent due to concerns regarding confidentiality, privacy, and low literacy levels. Given the sensitive nature of the questions, the interview process was immediately paused if any participant felt overwhelmed. Later, if needed, we provided psycho-social support (n = 2) by our trained team members and referred participants to the NIMH.

## Results

A total of 59 stakeholders participated in this study, of whom eight were gender specialists, six healthcare providers, and seven mental healthcare service providers. Gender specialists were from NGOs such as World Fish, Bangladesh Legal Aid and Services Trust (Blast), BRAC, Plan International, Fos Feminist, and Care Bangladesh, with 5–15 years of working experience in these sectors. The healthcare providers included a diverse range of professionals, such as pharmacy owners, community-level health workers, NGO coordinators, surveillance workers, and paramedical professionals. They had between 2 and 25 years of experience providing healthcare-related support within their communities. Mental healthcare providers were mostly senior psychologists specialised in educational and counselling psychology from MAYA Limited (online platform), BRAC University, Daffodil International University, the Women Support Initiative Forum (WSIF) (online platform), the Centre for the Rehabilitation of the Paralysed (CRP), and Brandix Apparel (Supplementary 2).

Moreover, seven male community leaders (age 41.3 ± 8.4 years, age range: 30–65 years), seven female community leaders (age 50.1 ± 7.7 years, age range: 35–65 years), 11 men residing in slums (age 38.5 ± 3.8 years, age range: 30–54 years), and 13 women residing in slums (age 35.3 ± 5.7 years, age range: 20–60 years) were also interviewed. Most females living in slums were aged 20–35, had no formal education, and worked as maids. Most husbands were rickshaw pullers and did not report perpetrating any abuse (Table 3). The male slum residents were primarily aged 31–40 and had completed secondary education (Table 3).

**Table 3:** Sociodemographic information of the male and female slum residents.

Type of stakeholders	Groups	Numbers
Women residing in slums		
Age range	20-35	6
	36-55	2
	>55	2
	Not reported	3
Educational qualifications	No formal education	6
	Primary education	3
	Secondary education	2
	Not reported	2
Occupation	Housewife	4
	Maid	5
	Garment worker	3
	Tailoring	1
Husbands’ occupation	Security guard	1
	Rickshaw puller	4
	Garment worker	1
	Business	3
	Clerical job	1
	Unemployed	3
Marital status	Married	11
	Separated within the last year	2
Living situation	One room with shared toilet and kitchen settings	13
Type of abuse reported	Physical abuse	1
	Verbal abuse	3
	None reported	9
Men residing in slums		
Age	20-30	3
	31 -40	5
	41-55	3
Occupation	Chef in a local restaurant	1
	Driver	3
	Carpenter	1
	Unemployed	1
	Shopkeeper	2
	Businessman	1
	Auto rickshaw driver	1
	Tailoring	1
Education	No education	1
	Primary education	4
	Secondary education	5
	Not reported	1

**Fig 1:**
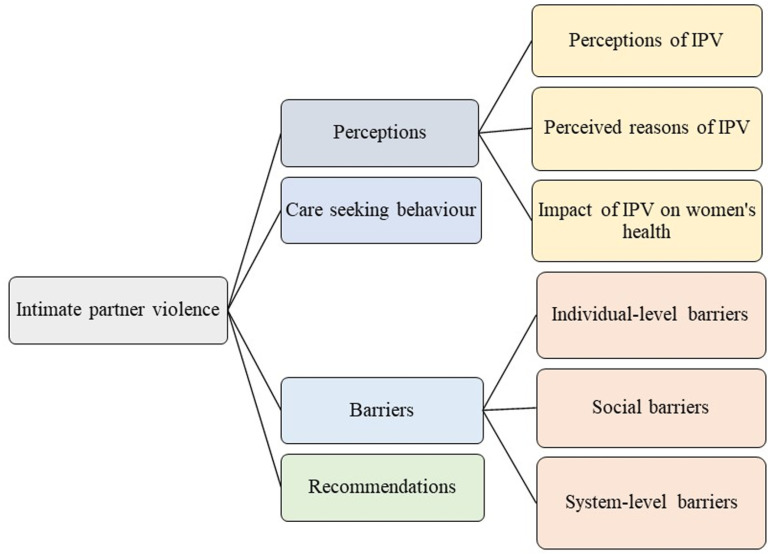
Themes and sub-themes.

Thematic analysis of the interviews with all stakeholders resulted in the identification of four main themes (see Fig 1), namely: 1) perceptions of IPV, 2) care-seeking behaviour among IPV victim-survivors, 3) perceived barriers in seeking mental healthcare, and 4) recommendations to address IPV and improve mental health services. These themes are presented below, accompanied by representative quotes from participants. We deliberately include a range of quotes from diverse stakeholders to amplify and uphold a variety of perspectives, ensuring all voices are centred and meaningfully represented.

### Theme 1: Perceptions of IPV: perceived reasons and impacts on women

All participants, including residents and care providers, shared their perceptions of IPV, discussing its prevalence in the community, the perceived reasons for its occurrence, and its overall impact on women.

Violence perpetrated by male intimate partners was considered an ordinary scenario among most women and men residing in slums. This was rooted in men's perceived authoritative position in the family as primary breadwinners, with violence against wives used as a form of correction or discipline. Female residents added that IPV is a part of their married life, often used by male partners as a weapon to make them abide by their rules. As a female resident stated:

*Our married life is full of arguments and fights. Whenever he is angry, he slaps me without even discussing the issues. It has become a common issue in my daily life. I think this happens to most women living in our slums.* CF-IDI-07

Male respondents perceived IPV as normal, particularly when their partners made what they considered to be unreasonable demands beyond their financial means. They also believed women needed to know and maintain certain courtesies with their husbands and in-laws. As one of them explained:

*Husbands can hit their wives who are irrational and always want things out of their economic ability.* CM-IDI-04

Some female community leaders echoed these thoughts, and one of them commented:

*If males do not get their demands fulfilled, they quarrel with their wives and manage their anger by hitting them. In our society, a man is titled a perfect husband when his wife fears him. Most women accept that their husband's abuse is their fate. Men’s negative behaviour towards their wives is always overlooked because of their gender role in society.* FL-KII-01

Gender and mental health specialists corroborated residents' perspectives. They agreed IPV is widely supported in Bangladeshi communities, mostly as a generational trend, especially in low-income populations. They mentioned that women often face abuse when they assert their needs or advocate for their rights. However, health specialists also mentioned IPV is common even in educated families who are aware of human rights. The acts of IPV are normalised, as people frequently witness IPV in their families. Due to prevalent cultural norms that are gender-based and place women in subordinated roles, IPV is accepted as a common occurrence for Bangladeshi wives. As illustrated by a mental healthcare service provider:

*IPV is easily accepted in our community, and hitting women has become habitual. Interestingly, IPV is common even in those people who are educated, aware of human rights, and know the health consequences of IPV. Regarding slum dwellers, they do not even consider it a crime as they have seen this happening in their own families as a trend in different generations.* MHP-FGD-04

This was supported by a gender specialist, noting that:

*Culturally, IPV is an established behaviour from a male partner. Even the women who are victims defend their husbands and accept the acts as culturally normative.* GST-KII-06

Under this main theme of perceptions, we found a set of reasons influencing IPV, which we report next as a sub-theme.

#### Perceived reasons for IPV.

When asked about the reasons for IPV, stakeholders indicated their varied perspectives, which were categorised into two major sub-themes: socio-cultural and interpersonal. Socio-cultural reasons included societal ideas about gender norms, early marriage, and dowry; interpersonal issues included a lack of awareness about IPV, unemployment, substance abuse, and extramarital affairs.

Most stakeholders indicated that the most profound reason for IPV among low-income populations is socio-cultural adherence to gender norms. According to gender specialists, people living in the slums have low socio-economic and educational status and tend to be more inclined to gender-specific behaviour structured by traditional cultural values. Participants also mentioned that women in slums are accustomed to tolerating IPV as they consider themselves lesser than men, internalising ideas of gender inferiority and subordination. The psychologists interviewed reinforced these statements on how the gendered role of men as sole family decision-makers influences the occurrence of IPV in slums. Healthcare providers added that although most women residing in slums contribute equally to household earnings, they seldom get the opportunity to exercise their decision-making rights, as men perceive such behaviour as a threat to their manhood. As one participant from Shyampur detailed:

*In slums, most wives don't have the freedom of speech. They are responsible for raising the kids and managing domestic duties. Most husbands are unwilling to contribute their income to the family, leaving women helpless. These women are then abused if they speak about their needs or oppose their husband's behaviour.* HCP-KII-04

According to some healthcare providers, the gendered practice of early marriage for girls further influences IPV. In low-income communities, most girls are married off during their adolescence period and are often too young to handle household responsibilities, which in turn creates household conflicts [62]. Due to the large age gap between girls/women and their husbands, they feel unable to voice their opinions or seek protection against violence. Gender specialists highlighted that because of younger ages and the possibility of recurrent violence, women residing in slums usually fear disclosing their issues to family or peers. As mentioned by a gender specialist:

*Early marriage is very common among slum women. They are often very young compared to their husbands and get beaten without any reason. Also, they continuously fear being abused again for complaining about these issues to their parents or relatives.* GST-KII-03

Additionally, some stakeholders saw some connection between IPV and dowries since:

*Most men abuse their wives for dowry. They force their wives to bring money from their [wife's] families. Failing to provide what they [husbands] want causes them to suffer immense physical or mental torture.* ML-KII-03

As added by an IPV survivor:

*My husband beats me when I oppose bringing money from my family*. CF-IDI-07

Interpersonal issues, also connected to gendered power imbalances in hetero-normative relationships, were perceived as reasons for IPV. As many husbands are unemployed, they forcefully ask their wives for money, abusing them physically and verbally. As one gender specialist disclosed:

*Some garment workers do not wish to pay their entire earnings to their husbands. Most of the time, their spouse beats them and forces them to give all their money. They come to us with bruises on their face and ask for advice on what they should do.* GST-KII-02

Stakeholders stated that unemployment contributes to the demand for dowry, substance use, and gambling among men residing in slums. One of the pharmacy owners explained:

*Most unemployed men in our community are addicted to substance use. They opt for gambling when they do not have money for these habits. Sometimes, they steal household money or emotionally abuse their wives to bring a dowry, which causes intimate partner violence.* HCP-KII-02

Substance abuse and gambling were further examples of interpersonal issues identified as contributing factors to IPV by both the women and the community leaders interviewed. Healthcare service providers agreed that some men waste household money on drugs and gambling instead of supporting their families. Women who protest these issues are often subjected to IPV.

Stakeholders from slums also highlighted extramarital affairs as an underlying interpersonal cause of IPV against women. For most community members, such issues frequently escalated to abusive behaviour by men, including grievous acts like homicides. As elucidated by a female community leader:

*Some men in our slums suspect their partners of having extramarital relationships. Lack of trust in spouses, suspicion of infidelity, and protesting husbands against polygamy are the common reasons for the IPV in our slums. On the other, despite having a wife, some men marry another woman without consent. When their first wife refuses to comply, their husbands end up causing them physical abuse.* FL-KII-05

Since interviews were conducted after the COVID-19 pandemic, participants were asked about its influence on IPV cases in the slums. According to most stakeholders, the incidence of IPV against women in the slums increased during the pandemic. Lengthy confinement in households, mobility restriction, and financial uncertainty were the perceived causes of IPV during the pandemic period. According to a female community leader:

*During the pandemic, people living in slums struggled to fulfil their basic needs, including losing their income source. People could not get out of their houses, which increased the cases of household conflicts, leading to different forms of IPV.* FL-KII-06

One of the male leaders concurred:

*I believe the issue of husbands abusing their wives increased during the COVID-19 pandemic. Most men employed in different daily wage works were compelled to stay at home. Also, they could not go to the stores and tea cafes where they used to meet their peers in the evenings, due to the lock-down. Staying at home and experiencing the challenges of the pandemic increased the tendency of intra-partner conflicts, leading to arguments, hostile voices, and, in most cases, physical abuse.* ML-KII-03

#### Impact of IPV on Women’s Health.

All stakeholders mentioned the negative consequences of IPV on women. Some underlined the physical health impacts on women who are assaulted, which could cause serious injuries. As expressed by a woman residing in a slum:

*My husband married me when I was only thirteen and started to hit me often. Previously, he already injured my limbs, which caused severe blood loss. Also, I have been hospitalised for some days for his extreme abuse. Remembering those days still traumatizes me.* CF-IDI-06

Thus, associated with those physical consequences are serious mental effects like trauma. Stakeholders described how IPV reduces the harmony of spousal relationships, thus increasing stress among women and indirectly impacting their children's healthy development. One mental healthcare provider explained:

*Experiencing IPV in a marital relationship is a deal breaker. Women who suffer from such issues are often under extreme distress and develop post-traumatic stress disorder. The impact on the children is much greater as it terrifies them when their mothers are scolded or beaten by their fathers* MHP-FGD-03.

Mental healthcare providers claimed that repeated experiences of IPV could increase suicidal behaviour among female slum residents, especially when they remain unaddressed for a long time. A psychologist said:

*A person who deals with IPV on a regular basis eventually becomes vulnerable to certain disorders such as anxiety, OCD, depression, PTSD, and even suicidal thoughts, because most of them are not seeking any care—neither for IPV nor for their mental health. They get into the vicious cycle of these issues.* MHP-FGD-07

Agreeing with this, one female slum resident disclosed

*Whenever my husband hits me, it hurts me to the core of my heart. I remain extremely upset and sad for days. I often get panic attacks and have tried to take my life when he abused me in front of other family members.* CF-IDI-10

Regarding other severe mental health impacts, a male community member revealed that his daughter was clinically diagnosed with severe depression and anxiety following repeated incidents of violence perpetrated by his son-in-law. He added:

*He [son-in-law] used to keep my daughter locked inside the house. She was not permitted to contact her family. He and his mother used to torment her physically and emotionally. Instead of taking care of my daughter, they used to complain to us about her poor well-being and lack of attention to household matters. Then I took her to the National Mental Health Institute for her unusual behaviour.* CM-IDI-08

Taken together, the perceptions, reasons, and impacts of IPV are consistent across all stakeholders: namely, that sociocultural and economic contexts affect—in complex and interconnected ways—female victim-survivors in Bangladeshi slums.

### Theme 2: IPV care-seeking behaviour among women victim-survivors

We explored the care-seeking behaviours of women living in slums in response to IPV and identified the key sources they most commonly rely on, as aggregated in this theme.

Most slum community members and leaders shared that when women affected by IPV seek support, it is firstly from informal sources such as older community members or leaders (e.g., landlords, local leaders, and other influential persons). Formal support seeking from healthcare or legal care providers is not very common, as indicated by all stakeholders. However, when women suffer from repeated and visible injuries that put their lives at risk, some female residents seek support from local police stations. One of the community leaders stated:

*Most IPV cases are resolved by elderly members of the slum who are well-known and respected in the community. They discuss the issues and try to reconcile between the husbands and wives. Additionally, there are internal slum committees that mediate disputes between spouses and wives about IPV instances. When female community members contact us for IPV assistance, we try to ensure justice for them.* FL-KII-05

In terms of IPV-related injuries or health concerns, stakeholders shared that the majority of women living in slums often fear sharing their IPV-related experiences with care providers. Seeking any formal support for verbal or sexual abuse is, thus, atypical. Nonetheless, healthcare providers at the slums disclosed that some female residents frequently seek assistance from local pharmacies for issues like stitches or painkillers related to physical injuries inflicted by IPV. However, they rarely share the source of their injuries with care providers. Women also visit the closest government hospitals or satellite clinics when injuries are severe, such as bone fractures or blood loss. Some female community members revealed that some men visit pharmacies to get medications for their spouses after hurting them. One of the female community members from Mirpur mentioned:

*When husbands severely injure their wives, they go to local pharmacies. Some fear letting the community know about their [wives] conditions; interestingly, their husbands buy medicines for them.* CF-IDI-04

When stakeholders were asked about mental healthcare seeking, most reported that women living in slums do not seek support from mental health professionals. People residing in slums supported this statement by noting that no mental health support services are available in their areas. Some also stated that women who experience violence by their husbands conceal their pain and do not express it to others, as the slum community can hold women responsible for the violence, labelling them as evil women (Kharap mohila). According to a male living in a slum:

*Often, women cannot express it or do not want to. The abuse continues in their homes. This is something that happens to many people, as I have seen. Even if it comes to light in some cases, women try to keep such information as private as they can. Sometimes, very serious incidents occur, and women need to be admitted to hospitals. These issues are often kept hidden as most people eventually blame females for the abuse they experienced. They call them kharap mohila [evil woman].* CM-IDI-10

One of the psychologists echoed that since the women are from a low socioeconomic background, it is difficult for them to seek support from psychologists. Yet, some women noted that when they experience low mood or suffer from psychosomatic issues like a lack of sleep, breathing difficulties, and frequent panic attacks due to their experiences of IPV, they visit local pharmacies or doctors to buy sleep medicine. Interestingly, one of the female community leaders claimed that IPV victim-survivors need their husbands' cooperation more than mental healthcare service providers:

*People residing in slums do not seek support for their mental health as this matter is unknown to them. Also, they have a little idea about the relevant support providers. Moreover, I believe these women need their husbands’ positive behaviour and emotional support more than any support from a professional.* FL-KII-02

In sum, despite different perspectives from stakeholders, we found common threads around how women seek informal and formal care. The next theme expands these threads by covering a range of barriers to seeking mental healthcare.

### Theme 3: Barriers to seeking mental healthcare

Stakeholder interviews revealed barriers for female slum residents seeking mental healthcare following an experience of IPV at individual, social, and system levels. Individual barriers are those influenced by women's perceived knowledge and awareness of the services. Social barriers include the effect of cultural and societal norms around IPV and gender roles. System-level barriers pertain to the gap in accessing services available by governmental organisations and NGOs.

#### Individual-level barriers.

Stakeholders noted that the main barrier to seeking mental health support for women experiencing IPV is having limited or no knowledge of mental health in general. Thus, they are unable to formally acknowledge their mental health difficulties or understand the role of mental healthcare services. Additionally, for some healthcare providers, most women have little knowledge about the mental health consequences of IPV. In fact, some people residing in slums mentioned that they were unaware of mental health and related care services. Accordingly, although some women residents experienced different mental health difficulties, they never sought care from formal mental healthcare providers. Instead, they chose to share their emotions regarding IPV with their family members and peers. As explained by one of the residents:

*I can go to a doctor when I feel ill, but I don't know what is mental health or how to understand these issues. I have seen my neighbours who have been tortured for more than ten years, but they also never seek care. Also, I had no idea there were any doctors or hospitals for mental health.* CF-IDI-03

*The concept of mental health does not exist in our community, as we often struggle to meet the basic ends for our family. Seeing a doctor for mere sadness seems a luxury to me. Also, most of us have no idea about where to go for mental healthcare services.* CF-IDI-03

Many people residing in the slums believed that doctors could not address mental health difficulties, as these issues are not as visibly apparent as general health problems. Consequently, they avoided disclosing their struggles to healthcare providers, instead confiding in trusted peers within the community who had similar experiences. As one female resident noted:

*How will a doctor treat my emotions? Doctors can only treat issues like fever or injuries. But they can never understand my inner pain; only my husband can help me overcome this situation.* CF-IDI-05

Similarly, another man residing in a slum stated

*I didn't know if there was a hospital to see a doctor for mental health. I thought one could only feel relieved by sharing one's emotions with others, such as friends and family members.* CM-IDI-06

#### Social-level barriers.

According to most stakeholders, fear and shame of disclosing mental health difficulties played a pivotal role in limiting mental healthcare-seeking behaviour. Most women residing in slums felt that sharing their emotional struggles with others often led to increased marital conflict within their families. They feared that their husbands might divorce them, labelling them as “mad” (“Pagol”). Additionally, there was a reluctance to disclose IPV to healthcare providers, as this could potentially result in legal repercussions for their spouses and in-laws. This was explained by a community health worker:

*Many women come to us with injuries on their faces perpetrated by their husbands. If we ask them to share their hidden emotions, they fear their husbands and in-laws will abuse them more. Also, due to the pre-conceived belief, the women will still consider their spouses as their masters and will try to keep them safe from any legal action by being quiet about their experience of IPV.* HCP-KII-05

Likewise, a mental healthcare service provider noted

*Despite being aware of its importance, even educated women feel uncomfortable seeking mental health support after experiencing IPV from any psychologist. So, female slum residents will feel more hesitant because of their perceived socio-cultural boundaries. Moreover, IPV and mental health are both taboos in our society; therefore, these issues remain unreported, particularly in low-income communities.* MHP-FGD-04

Some stakeholders further indicated that many women think revealing instances of IPV could bring social shame and judgment upon their extended families and communities. Gender specialists explained that IPV is often normalised in slum settings, and seeking informal mental health support—even from family members or relatives—can be seen as disgraceful, as it challenges a socially-accepted norm. Consequently, most women are reluctant to access IPV-related support, including mental health services, due to fear of negative responses from care providers. Internalised cultural beliefs that normalise IPV as a common experience among women, coupled with a lack of awareness about their rights and available support services, pose significant barriers to seeking care for IPV and mental health issues According to one gender specialist:

*Most of the slum women don't know about their rights. They have little idea about their right to get IPV-related support.* GST-KII-04

Thus, stakeholders contend that women endure abusive such behaviour to preserve their livelihood, social security, and their children's well-being and security/future. They fear losing their children's source of financial support or fracturing or breaking the family. So, they prioritise family stability over their own physical, mental, emotional, and sexual health, resorting to silence as a coping mechanism. These findings are illustrated by the quotes below, from a healthcare provider and female victim-survivor, respectively:

*Most women tolerate abusive behaviours as they fear losing financial security, and they think about their child's future. In our society, the life of a child whose parents separated is difficult. Most women do not receive child support after separation.* HCP-KII-04

*I think it is better to keep these issues (IPV) confidential. People would get to know and judge me if I said something about my husband to any doctor or any other family member. They would only blame me, as I was unable to compromise to save my marriage. If my husband hears that I have spoken about these issues with anyone else, he may stop giving me money or throw me out of the house. Thus, I don't seek any kind of support, including mental health. I have also seen other women doing the same in my community.* CF-IDI-10

Some psychologists perceive seeking mental healthcare as more challenging for women when they get divorced after experiencing IPV. They noted that there is a fear that speaking out will label them as mentally ill, justifying the abuse they endured suffered and even leading to marital separation, as noted by a stakeholder:

*Most females working in our organisation as garment workers who experienced IPV are uninterested in seeking mental health [support]. They share their issues with us and show interest in seeking support. However, they lose interest in seeking counsel due to the stigma and shame. They fear people might blame them for being mentally unstable, which is why they are abused by their husbands.* MHP-FGD-08

#### System-level barriers.

For stakeholders, mental health sector is especially underdeveloped in Bangladesh. Relatively few IPV-related support services are inclusive of mental healthcare facilities, which are mostly unknown and inaccessible to low-income communities. Some stakeholders (men residing in slums and healthcare providers) affiliated with NGOs mentioned that, while their organisation had planned to provide mental health services alongside their existing health and legal support services to IPV victim-survivors, they are still in the process of implementing those services.

*In Bangladesh, there is a huge need for mental health services, yet the system is mostly ignored. Only a few existing IPV-related support services provide mental health-related help; however, those are not accessible to female slum residents.* GST-KII-02

*I have previously worked in other NGO health facilities and a primary care facilities operated in slums. There is no NGO which arranges mental health support for IPV victim-survivors. Our organisation plans to provide such services, which might require more time.* HCP-KII-05

Healthcare providers also mentioned that the absence of awareness-raising initiatives to educate people living in slums about mental health contributes to the under-utilisation of available mental health support services. According to one healthcare provider:

*I have never seen any mental health-related awareness programs or campaigns in these areas. Since most people receive no education, they do not know where to seek mental health support after experiencing IPV.* HCP-KII-01

Most stakeholders confirmed that no community-based mental health services are available near slum areas. The lack of accessible services prevents many residents from learning about and accessing resources outside their communities, further limiting their understanding of their mental health needs. Only a few NGOs provide free-of-cost mental healthcare services through internationally funded projects; however, these are not permanently established in slum areas. As elucidated by a psychologist:

*I know that no established mental health services exist in slum areas. Some NGOs support IPV victim-survivors in slum areas, but these are not sustainable in the long run. Although the service is free, it is inadequate. We need to establish community-based services that are readily accessible and act as a continuum of care.* MHP-FGD-07

Some stakeholders, especially mental healthcare providers, highlighted the nationwide dearth of mental health professionals. This scarcity, along with the limited availability of service facilities, creates significant challenges for healthcare providers in making appropriate referrals, considering factors such as cost, transportation, medications, and therapies. Gender specialists further argued that community-level health workers in slum areas are not adequately trained to offer psycho-social support. Echoing this concern, a female resident remarked:

*Many Shashto Apa [community-level healthcare workers] come to inform and guide us about different health issues and family planning methods. However, no one ever came to hear about our emotional sufferings or to educate us about mental health.* CF-IDI-08

Similarly, a mental healthcare provider explained

*The number of mental health professionals is very low compared to the overall population in Bangladesh. Therefore, most people from slums cannot seek service. Due to the low number of service providers, most people don't know about them. This also contributes to the low knowledge of the slum population.* MHP-FGD-02

Nonetheless, gender specialists stated that

*The mental health needs of women living in urban slums are often unaddressed and untreated. IPV victim-survivors of the slums do not have mental health services in their areas where they can get support. In Dhaka city, women who report their cases to the police stations are asked to get help from the National Trauma Centre. Similarly, in district-level hospitals, some doctors and nurses are trained to provide emergency mental health counselling to women severely injured and battered by their intimate partners.* GST-KII-04

According to stakeholders, a lack of financial support was another substantial barrier to seeking mental health care. Women, who are often financially dependent on their husbands, frequently have their healthcare decision-making ability overlooked due to this dependency. The situation becomes even more challenging for IPV survivors, as requesting money to access mental healthcare can potentially provoke further abuse. As one mental healthcare provider explained:

*A woman who is an IPV survivor is already dominated by her husband for different issues. Seeking mental health support might be challenging, as she must maintain secrecy. If she is financially dependent on her husband, she would not be able to afford to get such services.* MHP-FGD-01

Women who are financially dependent also face difficulties in bearing the cost of mental healthcare services. Some community leaders noted that most women forgo even basic healthcare services, prioritising their children's essential needs, such as health and education, over their own. In line with this, a gender specialist emphasised that the overall cost of accessing mental healthcare services is prohibitive, encompassing provider fees, transportation, and medication costs:

*The residents of slums cannot afford private mental health services due to the high cost of such services. Public mental health services are affordable, but are not easily accessible due to distance and transportation costs.* MHP-FGD-08

As one of the male resident stated

*Often, financial constraints are one of the reasons—there might not be enough money, or sometimes, instead of seeking treatment, they keep aside the money for their children or other household expenses, neglecting their [own] care. It is also evident that there is a significant lack of awareness, as women are often not informed or conscious about these matters.* CM-IDI-06

Finally, healthcare providers stated that many women residing in slums might feel uncomfortable and unsafe due to the lack of privacy in their overcrowded households. A gender specialist further suggested that tele-counselling services could be a viable option for reaching low-income populations, as they are both accessible and affordable. However, ensuring privacy for clients remains a significant challenge, especially when they are living with their abusive partner.

*Online counselling services might be a convenient way to get mental health care, but it can be more difficult for women due to the lack of proper private and comfortable living in their houses.* GST-KII-05

Moreover, some participants added that the lack of regulatory mechanisms results in many counsellors failing to uphold proper ethical standards, which reduces the quality of care, negatively affects clients well-being, and discourages women with experiences of IPV from seeking support.

### Theme 4: Recommendations

While discussing barriers, most stakeholders shared insights to improve mental health support and services for IPV victim-survivors, which were categorised into three actionable recommendations: (1) increasing community-level awareness, (2) strengthening legal support, and (3) improving mental healthcare services.

#### Recommendation for increasing community-level awareness.

Most stakeholders indicated the urgency of initiating appropriate measures to reduce IPV cases in the community. Psychologists, healthcare providers, and community leaders emphasised organizing mass awareness-raising activities about IPV at the community level. They mentioned that intervention programmes that consist of participatory activities and behaviour change communication materials have the potential to educate men and women residing in slums about the types of IPV and their negative impact. Furthermore, community-based promotional activities, courtyard meetings, or digital media-based programmes through radio shows and television could circulate examples of positive masculine behaviour and IPV-related support services, eventually impacting perceptions and actions. According to one of the community leaders:

*Awareness programs should be implemented among slum dwellers to educate them about different aspects of gender-based violence. Different types of interventions such as role-playing, drama, and game-shows can help reduce conflicts among spouses.* ML-KII-04

As added by a male residents

*To raise awareness, we can distribute leaflets to households or gather the males of the community at a centre to have a conversion with them and provide counselling, if it could bring some benefits.* CM-IDI-04

Some stakeholders also stated that education at different institutional levels could be an effective way to increase awareness about domestic violence, as it would foster greater awareness of the impact of IPV on children living in slum areas who witness violence at home. One female resident stated:

*Educational curricula at school, college, and university levels should include content about domestic violence so that students can be aware early; eventually, the whole community could benefit...Most children living in slums witness IPV regularly. Therefore, all schools (particularly public) should take appropriate measures to educate these children; otherwise, some will develop similar behaviour to their surroundings.* CF-IDI-12

#### Recommendation for strengthening legal support and empowering women.

Stakeholders insisted on ensuring strong legal support and justice for IPV victim-survivors. They added that most IPV cases are under-reported due to an inadequate justice response, which seems to discourage care-seeking within the community. Women residing in slums insisted equality should be ensured while serving justice. Stakeholders noted that such an approach would also support the safeguarding of victim-survivors' mental health. Similarly, one of the men residing in the slums stated:

*We need to strengthen the existing legal actions against IPV perpetrators. I think these kinds of abusers should be jailed for a long time, and these instances should be disseminated to prevent future cases. The government should guarantee proper justice to all the victims of IPV.* CM-IDI-10

Additionally, female residents stated that women with experiences of IPV should be supported and empowered to become more financially independent from their partners. Financial assistance or opportunities for microcredit provided by the government could be effective initiatives. One of the male residents explained:

*A small loan can help female slum residents to start a small business. For example, 20,000–30,000 Taka could help her buy the raw materials for making paper bags, which she can sell in the local markets. Or, she can buy a sewing machine for 10,000 Taka, invest 4–5,000 Taka in training, and learn this work. With only 10–15,000 Taka, she can start making clothes. Thus, she can create a path to being independent in decision-making and financial terms and can break the vicious cycle of poverty and violence. I believe that if the government can provide this funding to every family, women can contribute to the household income.* CM-IDI-5

#### Recommendation to improve mental health services.

Stakeholders shared their perspectives on improving overall mental health services, which they contended must be inclusive for IPV victim-survivors. The majority insisted on increasing community-based intervention programmes so that people understand the importance of mental health, including the psycho-social needs of IPV victim-survivors. Further, it was argued that the government should extend funding of services to address the psychological needs of the low-income populations living in slums.

Stakeholders insisted that there needs to be institutional support to educate women about mental health and support women to be less economically reliant on their husbands. As highlighted by a female community member:

*We need community-based awareness activities to educate women about promoting their mental health, particularly following a traumatic experience in the family. These women need to learn basic self-care and maintain their quality of life. Moreover, the government should run empowerment activities so survivors are economically independent and can seek health care whenever necessary.* CF-IDI-13

For some stakeholders, strong commitments are needed from policymakers to integrate mental health services while providing primary healthcare to ensure easy access, mostly in emergencies, for people living in urban slums. People living in slums communicated that the presence of mental healthcare providers at urban primary care facilities would strengthen the care pathway of vulnerable women in the community. Similarly, health providers identified that placing psychologists at public hospitals would strengthen the referral system and ensure cost-effective mental health services for low-income populations. The psychologists interviewed for this study perceived that providing mental health for all people in slums could eventually reduce IPV cases:

*The government should exert more effort to provide easy access to mental healthcare services. Schools, garment factories, and offices should have at least one counsellor where people can seek support. Public hospitals should recruit psychologists besides doctors to generate appropriate referrals so that when women visit a doctor for violence-inflicted injuries, they can also seek mental health support to prevent complications.* HCP-KII-05

Some psychologists expressed the need to increase alternative mental healthcare providers by training paramedic professionals. Community leaders and inhabitants concurred that the healthcare workers placed in slums should be trained in basic mental health, as women residents tend to trust them. Agreeing with this, one healthcare worker shared:

*We visit many houses and counsel women about different health issues on a regular basis. If volunteers like us are trained to screen and refer for mental health issues, it will help to create awareness, eventually strengthening the care pathway for female victim-survivors of domestic violence. Eventually, this can sensitize the entire slum population.* HCP-KII-02

In this context, mental healthcare providers proposed the creation of self-help groups of women through peer networks, including IPV victim-survivors of the slums. As noted by gender specialists, men living in slums should also be included in IPV-focused social interventions and programmes. As illustrated by a psychologist:

*We can arrange mental health awareness and self-care workshops in slum communities. Both the male and female victim-survivors can attend these workshops. They can share their problem in group settings and find a mechanism to support each other. The presence of male dwellers can help sensitise their peers and ensure future support programmes’'sustainability.* MHP-FGD-06

For some stakeholders, technology-based support services could help disseminate proper IPV and mental health-related information. Mobile phones and social media platforms are easily accessible to the slum population and can help contribute to mass mental health awareness activities [63]. Psychologists explained that online mental healthcare services could eventually resolve the challenges regarding the low number of service providers by reducing the time, cost, and distance-related barriers. Women who experience IPV can access these mental healthcare services in emergencies anytime through their mobile phones. Helpline or hotline numbers where they can access support for IPV and mental health counselling should be further disseminated in slums. One of the gender specialists stated:

*We can use technology-based platforms for the widespread circulation of information related to domestic violence and mental health. Most slum dwellers know the application of mobile texts, Facebook, WhatsApp, IMO etc. We can develop relatable Bengali content so they can understand the information easily.* GST-KII-02

Similarly, another specialist added

*We have to aware people of the available services so that they access those in need. We must share positive stories about IPV victim-survivors who received mental health support or counselling. We can encourage the community to promote their mental healthcare-seeking behaviour through these activities.* GST-KII-06

To conclude, these recommendations stakeholders recommended a multi-pronged approach to support IPV victim-survivors in slums, including: raising community awareness about IPV and mental health; strengthening legal support for IPV victim-survivors; financially empowering women in slums; integrating mental health services into primary care; training mental healthcare providers; and leverage technology to improve access to information and support services.

## Discussion

Globally, intimate partner violence is a major societal issue and a recognised public health concern impacting individuals, families, and communities. IPV has largely been conceptualised as a human rights violation and can have various adverse physical and emotional health impacts [3]. Previous research has explored the mental healthcare-seeking behaviour of female victim-survivors of IPV in the Global North and has identified some key associated factors such as ethnicity, cultural norms, clinical environment, service users’ interest, and availability of resources [22,64]. Limited studies have been undertaken with marginalised low-income communities in the Global South, such as in Bangladesh, where there is a paucity of mental health services. To the best of the authors' knowledge, this is the first study to explore the perceptions of IPV and patterns of mental healthcare-seeking among women living in urban slums. The findings highlight universal challenges in seeking mental healthcare, along with unique barriers faced by female victim-survivors of IPV living in slums. Stakeholders also voiced concerns about different social and systemic barriers to accessing mental healthcare. They suggested potential strategies to strengthen care pathways and better safeguard women who experience violence. These insights carry significant implications for community outreach, education, and the necessity for further research to enhance policy practices.

Our findings demonstrate the social aspect of IPV in low-income communities of Bangladesh by unveiling that some forms of violence are perceived by all stakeholders as acceptable. They noted IPV is common and even normalised in most Bangladeshi communities, including in urban slums. Perceived gender differences characterised by cultural beliefs and social norms appear to influence gender-specific roles in the slum community: men are believed to be more powerful and superior to women. This dynamic typically places men in control of household decision-making, subordinating women and increasing their risk to IPV [8]. A recent study in Zimbabwe similarly indicated IPV is sustained by societal ideas that legitimise violence, accept male dominance, and stigmatise women who report abuse [20].

In addition to considering the influence of gender norms on IPV occurrence, our study further explored different types of interpersonal and sociocultural contributors to IPV in slums, such as a lack of awareness, financial insecurity, early marriage, dowry practices, substance abuse, and extramarital affairs. The association of these factors with IPV was also highlighted in previous studies conducted in developing countries [65–69]. A WHO multi-country study conducted in 11 countries, including Bangladesh, Brazil, and Ethiopia, corroborated that alcohol abuse, attitudes supporting wife beating, affairs, and growing up with domestic violence increased the risk of IPV [14,70–73]. Our study also documented the influence of the COVID-19 pandemic on IPV. Consistent with previous studies [10,68,74], factors including household confinement, mobility restrictions, increased household stress, and economic insecurity were associated with higher rates of IPV during the pandemic.

Regarding care-seeking behaviour, stakeholders highlighted that IPV victim-survivors often face barriers to accessing immediate legal and healthcare assistance, increasing their vulnerability to severe physical and mental health consequences that are likely to persist over time. Seeking informal support from their community leaders or influential persons following abuse from their husbands was the most familiar practice among women residing in slums. In terms of system-based care-seeking, female residents occasionally sought help from local police stations in cases of recurrent abuse or inflicted injuries. However, stakeholders noted that such efforts were often hindered by fear and a reluctance to disclose IPV-related issues within their close-knit community, ultimately leading many to rely on informal support rather than formal care [18,75]. Our findings also underscored that such attitudes represent a substantive social-level barrier to accessing mental healthcare services. Evidence indicates that this exacerbates the mental health challenges faced by victim-survivors [28]. As observed in other Global South countries, embarrassment and fear of adverse consequences following disclosure often prevent individuals from seeking community, legal, or medical support [17,75–77]. Moreover, the dual stigma of experiencing IPV and living with mental health conditions further discourages the seeking of formal mental healthcare [78]. These factors interact bidirectionally, contributing to IPV against women and potentially hindering the acceptance of interventions [79].

In addition to these social hinderances, our study identified individual and system-level barriers that interplay with mental healthcare-seeking pathways among female IPV victim-survivors from urban slums. At the personal level, lack of mental health literacy among IPV victim-survivors is a significant barrier to care seeking, consistent with findings from studies from other developing settings [58,75]. Stakeholders reported that women tend to prioritise the physical impacts of IPV rather than focusing on psychological effects [59,80,81]. Low educational status and financial dependency of slum women might contribute to such issues [59,79,82], however, studies have demonstrated that higher education is closely related to better recognition of health problems and awareness of human rights [83].

At the system level, the absence of community-based mental health services, dearth of mental health specialists, shortage of trained community-level health workers in mental health, and poor referral procedures were mentioned by stakeholders as critical barriers. Other studies have identified similar issues, including low focus on the psycho-social aspects of health and a lack of effective referral systems [59,80]. Inadequate mental health literacy programs and the absence of facilities in primary healthcare facilities might significantly impact the mental healthcare seeking of slum residents, including IPV victim-survivors [84,85].

Currently, formal support services for gender-based violence at informal settlements are provided temporarily through time sensitive projects, mainly by NGOs in Bangladesh. The multi-sectoral program on violence against women is led by the Ministry of Women and Children Affairs (MOWCA) of Bangladesh through One Stop Crisis Centres (OCC) and the National Trauma Counselling Centre (NTCC) in divisional areas [46]. OCCs are based at tertiary-level healthcare facilities (government-level medical colleges) and provide health care, police assistance, DNA tests, social services, legal assistance, psychological counselling, and shelter services to women victim-survivors. NTCC is the government-level specialised health centre that provides psycho-social counselling and related support to women and children who are victim-survivors of domestic violence, although the number of NTCCs is very low compared to the population. However, due to low knowledge of available services among slum women, these services are seldom accessed. Factors such as inadequate resources, community propensity for victim blaming, high acceptability of violence, and a proclivity to overlook violence might also diminish the support-seeking attitude of IPV victim-survivors [18,86].

The factors influencing IPV are multifaceted, with significant physical and mental health impacts not only on victim-survivors, but also on children, as IPC leads to “inter-generational transmission of violence,” enhancing the probability of perpetration (by males) and victimisation (females) in adulthood [70–73]. Our study participants provided extensive suggestions to alleviate the challenges related to IPV and mental health. Based on their suggestions and grounded in evidence from similar countries settings, we derived the following recommendations:

A. ***Community based interventions:*** Stakeholders emphasised the importance of community-based interventions featuring participatory activities and behaviour change communication (BCC) materials to raise awareness about the types and impacts of IPV. They also recommended including emergency helpline numbers as part of awareness campaigns to help create a protective environment within slum communities. All stakeholders also believed that creating self-help groups of IPV victim-survivors was another critical strategy. These interventions were reported to help reduce IPV cases and ensure time-sensitive psycho-social first aid, effective referrals, access to social support, improved mental health outcomes, and enhanced safety behaviours of victim-survivors [87]. In fact, a clustered randomised control trial on reducing IPV cases in Bangladesh indicated the effectiveness of participatory group sessions and community mobilisation through BCC materials such as poster distribution, street drama, banner campaigns, and reflective dialogue [88].

Community-based peer group discussions can also inspire victim-survivors to seek care for IPV [89]. Women with prior lived experience of IPV can be included in designing a peer support model to assist their peers in understanding coping mechanisms; evidence supports that such programs improve self-esteem, well-being, and resilience [80,90,91]. Furthermore, key stakeholders, family members, and male community members can be engaged in implementing community-based interventions that are culturally tailored, gender-transformative, and couple-centred [80,90,91]. Such interventions can potentially cultivate healthier, more equitable spousal relationships, shift household dynamics, and promote non-violent communications, thus contributing to improved well-being [90,91].

B. ***Healthcare system improvements:*** Stakeholders urged relevant authorities to prioritise implementing mental health policies and finance the design and implementation of sustainable and cost-effective mental health interventions. Increasing alternative mental healthcare provision by training doctors, paramedics, and other healthcare workers as psycho-social first-aid support providers has the potential to ensure mental health services for all [91,92]. Informal support providers such as pharmacists living in slum areas, female community leaders, and traditional and faith healers can be turned into mental health task forces through educational interventions, which can further contribute to the cause [73,93]. Adding to this, stakeholders also highlighted the importance of accessibility to affordable mental healthcare services, which can be ensured by integrating them into urban primary care with digital availability and promoting existing government and NGO resources [85,94]. Global studies found suggestions comparable with our findings mitigated the challenges of providing mental healthcare in LMIC settings like Bangladesh [95,96]. Moreover, IPV screening and related mental healthcare can be incorporated into routine clinical care and perinatal mental health services, as these were successful in similar settings, such as Kenya and India [97–99]. Such initiatives are achievable within a short period and will be helpful avenues for ensuring the well-being of survivors by providing basic mental health support and needed referrals for severe cases [73,93,97–99].
C. **Institutional responses:** Stakeholders stressed the importance of ensuring legal support to uphold women's rights to fight against IPV and its consequences. In Bangladesh, the Domestic Violence (Prevention and Protection) Act 2010 created an opportunity for women to receive justice, but many do not know it exists or understand its impacts [16]. To successfully respond to domestic violence and protect victim-survivors while holding abusers accountable, an Indian study suggested capacity building of police, legal professionals, protection officers, and social and healthcare workers to enhance their abilities in these areas [100]. Special training of law enforcement agencies on available legal provisions to address violence against women might improve the uptake of formal social and healthcare services [16]. Also, programs implemented in Uganda that promote institutional partnerships among law enforcement forces, government healthcare facilities, and community-level organisations created a pathway to provide trauma-informed support practices to support the mental health of IPV victim-survivors [92,101]. With government and non-government collaborative funding, specialised facilities such as Thuthuzela Care Centres in South Africa and Gender-Based Violence Recovery Centre in Kenya can be stationed beside settlements to ensure comprehensive and accessible legal, health, and mental healthcare [102]. Thus, relevant stakeholders should prioritise preventive efforts against IPV by properly implementing existing policies, enforcing laws, and supporting advocacy for establishing a human rights viewpoint of women that can help ensure a gender-based-violence-free society.
D. **Prevention and Awareness Campaigns:** Stakeholders recommended using digital media to promote positive masculine behaviour and conducting educational programs to inform slum residents about IPV-related support services. An early formal education curriculum should also include basic SRH rights and mental health knowledge, which would benefit both males and females at the beginning of their childhood. School-based programs like SASA in Kampala and South Africa were found feasible in slum settings in explaining the consequences of IPV and its impact on the mental health of young people [103]. Moreover, evidence supports that community-based dialogues and male-focused IPV workshops emphasising healthy masculinity and partner support to maintain respectful relationships were promising in slums [33,103,104]. Also, various media outlets such as radio and TV programmes can play an integral role in increasing mass awareness against IPV at the national level [94,105–107]. Incorporating mobile phone and social media-based messages may reach vulnerable women who rely on informal networks [63,108–110].
E. ***Government-level initiatives in the long term:*** Given the multitude of challenges and impact of IPV on women and society, a multi-sectoral response is required to tackle these challenges in the long term. Such a response must combine development activities like improving access to education—with initiatives to transform gender norms and attitudes and to create employment opportunities that promote women's empowerment [14]. Subsidised care for IPV victim-survivors (i.e., India’s Nirvaya fund), economic empowerment programs involving micro-financing or vocational training, and occupational rehabilitation to foster women's safety, financial autonomy, reducing dependence on partners and enhancing the opportunities for women to seek proper care for IPV [111]. Simultaneously, the promotion of male education, employment, and substance abuse programs would play a pivotal role in nurturing economic stability and hindering root causes. Lastly, to inform policymakers and related stakeholders about the overall situation of IPV, it is crucial to create a central data collection system for tracking IPV data and related psychological needs.

## Strengths and limitations

A major strength of our study was the variety of stakeholders included and the qualitative design that allowed us to capture in-depth and rich perceptions and experiences of IPV and mental health across different groups. Nonetheless, the study was based on a convenience sample and some linguistic and semantic issues may have been lost during the translation of interviews from Bangla to English. Although the qualitative study approach constrains generalisability, these findings may be highly pertinent to other low-income contexts.

## Conclusion

The study presented a variety of concerns affecting access to mental health care by IPV victim-survivors, namely low mental health literacy, gender norms, social stigma, financial dependence, limited awareness of services, and lack of professionals and resources. To foster equal access throughout urban slums, stakeholders emphasised the significance of better and more accessible mental health services, training of non-specialists, and the need to foster referral networks. It would be possible to promote mental health literacy by increasing public knowledge of IPV and mental health issues as well as by promoting accessible services through official government health education initiatives. Stakeholders also demanded strengthening violence prevention efforts through community-based interventions, encouraging healthy interpersonal relations and a stronger commitment to social justice.

## Supporting information

S1 File Qualitative guidelines. (DOCX)

S1 Table Identification of the stakeholders. (DOCX)

S1 Checklist COREQ (Consolidated criteria for Reporting Qualitative research) Checklist. (DOCX)
