# Supplementary material for: Exploring the pattern of mental health support-seeking behaviour and related barriers among women experiencing intimate partner violence in urban slums of Bangladesh: perspectives from multiple level stakeholders
Source: PLOS Glob Public Health. 2025 May 9;5(5):e0004568. doi: 10.1371/journal.pgph.0004568 (PMC12063865; doi:10.1371/journal.pgph.0004568)
Supplement: S1 File — (DOCX) [file pgph.0004568.s001.docx]

**S1 File: Qualitative guidelines**

**In depth interview guidelines: Female members of the community/ survivor of IPV (Bangla)**

**1. Socio-demographic Information:**

- বয়স
- লিঙ্গ
- শিক্ষাগত যোগ্যতা
- পেশা

**2. Experience of IPV:**

- আপনি কি আমাকে আপনার স্বামীর দ্বারা সহিংসতার সম্মুখীন হওয়ার ঘটনা সম্পর্কে বলতে পারেন? কারণ টা কি ছিল?
- আপনি কি আপনার উপর নারীর প্রতি স্বামীর দ্বারা সহিংসতা/IPV এর প্রভাব সম্পর্কে আমাদের বলতে পারেন?

·        শারীরিক

·        মানসিক

- আপনি কি মনে করেন যে নারীর প্রতি স্বামীর দ্বারা সহিংসতা/IPV মানসিক স্বাস্থ্যের উপর উল্লেখযোগ্য প্রভাব ফেলে? যদি তাই হয়, দয়া করে আমাদের বলুন কিভাবে তারা সম্পর্কযুক্ত।

**3. Information related to IPV:**

- আপনার এলাকায় কোন ধরনের নারীর প্রতি স্বামীর দ্বারা সহিংসতা/IPV বেশী দেখা যায়? সমাজের নারীর প্রতি স্বামীর দ্বারা সহিংসতা/IPV হওয়ার পিছনে কি কোন কারন আছে?
- শারীরিক
- যৌন
- মানসিক
- আপনার চারপাশে কত ঘন ঘন নারীর প্রতি স্বামীর দ্বারা সহিংসতা/IPV ঘটে? আপনার এলাকায় নারীর প্রতি স্বামীর দ্বারা সহিংসতা/IPV এর তীব্রতা (কতটা) সম্পর্কে আপনি কি আমাদের কিছু বলতে পারেন?

**4. Individuals/Family / household-level influences:**

- আপনি কি মনে করেন যে পুরুষ এবং মহিলার বয়স ও পারিবারিক অবস্থা নারীর প্রতি স্বামীর দ্বারা সহিংসতা/IPV এর সাথে সম্পর্কিত? যদি হ্যাঁ হয়, কিভাবে?
- আপনি কি মনে করেন যে পুরুষদের ছোটবেলার কষ্টদায়ক স্মৃতি অভিজ্ঞতা নারীর প্রতি স্বামীর দ্বারা সহিংসতা/IPV এর জন্য দায়ী? যদি তাই হয়, দয়া করে ব্যাখ্যা করুন।
- পুরুষ ও মহিলাদের শিক্ষাগত যোগ্যতা এবং নারীর প্রতি স্বামীর দ্বারা সহিংসতা/IPV এর মধ্যে কি কোন সম্পর্ক আছে? যদি হ্যাঁ হয়, কিভাবে?
- আপনি কি মনে করেন বাল্যবিবাহ এর সাথে নারীর প্রতি স্বামীর দ্বারা সহিংসতা/IPV সম্পর্কযুক্ত? কিভাবে দয়া করে ব্যাখ্যা করুন।
- আপনার কি মনে হয় পুরুষের বেকারত্ব নারীর প্রতি স্বামীর দ্বারা সহিংসতা/IPV ঘটার একটা কারন? যদি তাই হয়, আপনার মতামত জানান।
- আপনি কি মনে করেন যে পুরুষের জুয়া খেলা, নেশাদ্রব্য গ্রহন বা বিবাহ বহির্ভূত সম্পর্কে জড়ানো নারীর প্রতি স্বামীর দ্বারা সহিংসতা/IPVর জন্য দায়ী?
- আপনি কি মনে মহিলাদের ঘরের বাহিরে কাজ করা তাদের বিরুদ্ধে সহিংসতাকে বাড়িয়ে তোলে? দয়া করে বিস্তারিত বলুন।
- যেহেতু আমরা কভিড-১৯ এর মধ্য দিয়ে যাচ্ছি, আপনি কি মনে করেন কভিড-১৯ এর সাথে নারীর প্রতি স্বামীর দ্বারা সহিংসতা/IPV এর কোন সম্পর্ক আছে? আপনি কি আমাদের কিছু উপাদান সম্পর্কে বলতে পারবেন যেগুলো কভিড ১৯ এর সময়ে নারীর প্রতি স্বামীর দ্বারা সহিংসতা/IPVর পিছনে দায়ী?
- খাবারের যোগাড় এবং ক্রয়ক্ষমতা
- কর্মসংস্থানের অবস্থা
- আর্থিক সাহায্য
- কোভিডে আক্রান্ত হওয়ার ভয়

**5. Community-level influences:**

- আপনি কি মনে করেন যে সমাজের বিভিন্ন সহিংস ঘটনা পুরুষদের এই ধরনের আচরণ শিখতে এবং করতে উত্সাহিত করে? যদি তাই হয়, দয়া করে ব্যাখ্যা করুন।
- আপনি কি মনে করেন যে তালাকপ্রাপ্ত মহিলাদের প্রতি সমাজের যে নেতিবাচক ধারনা রয়েছে তা একটি অস্বাস্থ্যকর বা আপত্তিজনক সম্পর্কে থাকার কারণ?

**6. Current IPV support services**

- বর্তমানে নারীর প্রতি স্বামীর দ্বারা সহিংসতা/IPV এর জন্য যে সাহায্য ও সেবা রয়েছেতা সম্পর্কে আপনি জানেন কি যদি হ্যাঁ হয় তবে, আপনি কি এই সাহায্যগুলো নিয়েছেন ?
- বাংলাদেশের বর্তমান নারীর প্রতি স্বামীর দ্বারা সহিংসতা/IPV সংক্রান্ত পরিসেবা সম্পর্কে আপনার মতামত কি?
- স্তর
- গুনমান
- পরিমান (নারীর প্রতি স্বামীর দ্বারা সহিংসতা/IPV সংগ্রামীদের জন্য কোন ধরনের সেবা পাওয়া যায়?সব জায়গায় কি সেবা গুলো পাওয়া যায়?)
- আপনি নারীর প্রতি স্বামীর দ্বারা সহিংসতা/IPV সহায়তা পাওয়ার ক্ষেএে কি কি বাধার সম্মুখীন হয়েছেন? কিধরনের বাধার সম্মুখীন হয়ছেন দয়া করে ব্যাখ্যা করুন ।
- সামাজিক
- সাংস্কৃতিক
- অর্থনৈতিক
- সচেতনতা ও শিক্ষা সম্পর্কিত
- কলঙ্ক ও ভয়

**7. Recommendations:**

- নারীর প্রতি স্বামীর দ্বারা সহিংসতা/নারীর প্রতি স্বামীর দ্বারা সহিংসতা/IPV কিভাবে কমানো যায় এই সম্পর্কে আপনার কোন পরামর্শ আছে? যদি থাকে আমাদের বলুন।
- নারীর প্রতি স্বামীর দ্বারা সহিংসতা/নারীর প্রতি স্বামীর দ্বারা সহিংসতা/IPV সহায়তা চাওয়ার সাথে যুক্ত বাধাগুলো কমাতে কি করা যেতে পারে? আপনার মতামত জানাবেন।

**In depth interview guidelines: Female members of the community/ survivor of IPV (English)**

**1. Socio-demographic Information:**

- Age
- Gender
- Educational status
- Occupation

**2. Experience of IPV:**

- Can you tell me about the incidence when you experienced violence by your husband?
- What was the reason?
- Can you please tell us the effect of IPV on you?
- Physical
- Psychological
- Do you think that IPV has a significant impact on mental health? If so, please tell us how they are connected

**3. Information related to IPV:**

- What kind of IPVs are commonly faced by women around you? Is there any reason behind the prevalence of particular type of IPV?
- Physical
- Sexual
- Emotional
- How frequently IPV happens around you? Can you tell us something about the severity of IPV?

**4. Individuals/Family / household-level influences:**

- Do you think that age and family status of the male and female are related to the perpetration of IPV? If yes, how?
- Do you think that men’s adverse childhood experiences are responsible for perpetrating IPV? If so, please explain.
- Is there any connection between male’s and female’s education and perpetrating IPV? If so, please explain.
- Is child marriage related to IPV? If so, please explain.
- Do you think male’s unemployment is a reason of happening IPV?
- Do you think man’s involvement in gambling or substance abuse or extramarital relationship/sex is related to IPV? Please explain.
- Do you think that women’s open opportunity in income generating activities triggers violence against themselves?
- As we are going through Covid-19 pandemic, do you think that Covid-19 has a connection with increased IPV? Can you please tell us about the factors that are responsible for IPV during Covid-19?
- Food availability, accessibility &affordability
- Employment status
- Financial aid (what/ whom)
- Anxiety due to uncertainty of being affected

**5. Community-level influences:**

- Do you think widespread violence in the community encourages men to learn and practice violent behaviour? If so, please explain.
- Do you think that negative attitudes towards divorced women is a reason for staying in an unhealthy or abusive relationship?

**6. Current IPV support services**

- Do you think women know about the current IPV support services and receive them when necessary?
- What is your opinion regarding current IPV support services in Bangladesh?
- Level
- Quality
- Quantity: What services are available to IPV survivors seeking help? Are they available in all areas?
- Sector involved
- What are the challenges or faced by the victim of IPV in terms of receiving IPV support services?
- Social
- Cultural
- Economical
- Awareness and education related
- Stigma and Fear

1. **Recommendations:**

- Do you have any suggestion on how IPV could be reduced? If so, Please tell us briefly.
- What can be done to reduce the challenges associated with seeking IPV support?

**Key Informant Interview Guidelines: Male community members/ Male community leaders /Female community leaders/**

**1. Socio demographic information**

- বয়স
- লিঙ্গ
- শিক্ষাগত যোগ্যতা
- পেশা

**2. IPV related information:**

আপনার এলাকায় কোন ধরনের নারীর প্রতি স্বামীর দ্বারা সহিংসতা/IPV বেশী দেখা যায়? সমাজের নারীর প্রতি স্বামীর দ্বারা সহিংসতা/IPV হওয়ার পিছনে কি কোন কারন আছে?

- শারীরিক
- যৌন
- মানসিক
- আপনার চারপাশে কত ঘন ঘন নারীর প্রতি স্বামীর দ্বারা সহিংসতা/IPV ঘটে? আপনার এলাকায় নারীর প্রতি স্বামীর দ্বারা সহিংসতা/IPV এর তীব্রতা (কতটা) সম্পর্কে আপনি কি আমাদের কিছু বলতে পারেন?

**3. Individuals/Family / household-level influences:**

- আপনি কি মনে করেন যে পুরুষ এবং মহিলার বয়স ও পারিবারিক অবস্থা নারীর প্রতি স্বামীর দ্বারা সহিংসতা/IPV এর সাথে সম্পর্কিত? যদি হ্যাঁ হয়, কিভাবে?
- আপনি কি মনে করেন যে পুরুষদের ছোটবেলার কষ্টদায়ক স্মৃতি অভিজ্ঞতা নারীর প্রতি স্বামীর দ্বারা সহিংসতা/IPV এর জন্য দায়ী? যদি তাই হয়, দয়া করে ব্যাখ্যা করুন।
- পুরুষ ও মহিলাদের শিক্ষাগত যোগ্যতা এবং নারীর প্রতি স্বামীর দ্বারা সহিংসতা/IPV এর মধ্যে কি কোন সম্পর্ক আছে? যদি হ্যাঁ হয়, কিভাবে?
- আপনি কি মনে করেন বাল্যবিবাহ এর সাথে নারীর প্রতি স্বামীর দ্বারা সহিংসতা/IPV সম্পর্কযুক্ত? কিভাবে দয়া করে ব্যাখ্যা করুন।
- আপনার কি মনে হয় পুরুষের বেকারত্ব (চাকুরি না থাকা) নারীর প্রতি স্বামীর দ্বারা সহিংসতা/IPVর একটা কারন? যদি তাই হয়, আপনার মতামত জানান।
- আপনি কি মনে করেন যে পুরুষের জুয়া খেলা, নেশাদ্রব্য গ্রহন বা বিবাহ বহির্ভূত সম্পর্কে জড়ানো নারীর প্রতি স্বামীর দ্বারা সহিংসতা/IPVর জন্য দায়ী?
- আপনি কি মনে মহিলাদের ঘরের বাহিরে কাজ করা তাদের বিরুদ্ধে সহিংসতাকে বাড়িয়ে তোলে? দয়া করে বিস্তারিত বলুন।

**4. Intensity of the problems**

- যেহেতু আমরা কভিড-১৯ এর মধ্য দিয়ে যাচ্ছি, আপনি কি মনে করেন কভিড-১৯ এর সাথে নারীর প্রতি স্বামীর দ্বারা সহিংসতা/IPV এর কোন সম্পর্ক আছে? আপনি কি আমাদের কিছু উপাদান সম্পর্কে বলতে পারবেন যেগুলো কভিড ১৯ এর সময়ে নারীর প্রতি স্বামীর দ্বারা সহিংসতা/IPVর পিছনে দায়ী?
  - খাবারের যোগাড় এবং ক্রয়ক্ষমতা
  - কর্মসংস্থানের অবস্থা
  - আর্থিক সাহায্য
  - কোভিডে আক্রান্ত হওয়ার ভয়

**5. Community-level influences**

- আপনি কি মনে করেন যে সমাজের বিভিন্ন সহিংস ঘটনা পুরুষদের এই ধরনের আচরণ শিখতে এবং করতে উত্সাহিত করে? যদি তাই হয়, দয়া করে ব্যাখ্যা করুন।
- আপনার মতে যে ধরনের মনভাব সহিংসতাকে মেনে নেয় এবং লিঙ্গবৈষম্যকে শক্তিশালী করে তোলে, তা কি নারী সহিসতার জন্য দায়ী? যদি হয়, আপনার নিজের ভাষায় ব্যাখ্যা করুন।
- আপনি কি মনে করেন যে তালাকপ্রাপ্ত মহিলাদের প্রতি সমাজের যে নেতিবাচক ধারনা রয়েছে তা একটি অস্বাস্থ্যকর বা আপত্তিজনক সম্পর্কে থাকার কারণ?

**6. Relationship of IPV and mental health**

- আপনি কি দয়া করে আমাদের বলতে পারেন নারীর প্রতি স্বামীর দ্বারা সহিংসতা/IPV মহিলাদের উপর কী ধরনের প্রভাব ফেলে?
- শারীরিক
- মানসিক

আপনি কি জানেন যে নারীর প্রতি স্বামীর দ্বারা সহিংসতা/IPV মানসিক স্বাস্থ্যের উপর উল্লেখযোগ্য প্রভাব ফেলে? যদি তাই হয়, দয়া করে ব্যাখ্যা করুন।

**7. Current mental health support for IPV survivors**

- আপনি কি মনে করেন যে মহিলারা বর্তমান নারীর প্রতি স্বামীর দ্বারা সহিংসতা/IPV সমর্থন পরিসেবা সম্পর্কে জানে এবং প্রয়োজনমত সেবা গ্রহন করে?
- বাংলাদেশের বর্তমান নারীর প্রতি স্বামীর দ্বারা সহিংসতা/IPV সংক্রান্ত পরিসেবা সম্পর্কে আপনার মতামত কি?
- স্তর
- গুনমান
- পরিমান (নারীর প্রতি স্বামীর দ্বারা সহিংসতা/IPV এর শিকার মহিলাদের জন্য কোন ধরনের সেবা পাওয়া যায়?সব জায়গায় কি সেবা গুলো পাওয়া যায়?)

**8. Barrier**

- নারীর প্রতি স্বামীর দ্বারা সহিংসতা/IPV সহায়তা পাওয়ার ক্ষেএে নারীর প্রতি স্বামীর দ্বারা সহিংসতা/IPV এর শিকার ব্যক্তিরা কি কি বাধার সম্মুখীন হন?
- সামাজিক
- সাংস্কৃতিক
- অর্থনৈতিক
- সচেতনতা ও শিক্ষা সম্পর্কিত
- কলঙ্ক ও ভয়

1. **Solution:**

- নারীর প্রতি স্বামীর দ্বারা সহিংসতা/IPV কিভাবে কমানো যায় এই সম্পর্কে আপনার কোন পরামর্শ আছে? যদি থাকে আমাদের বলুন।
- নারীর প্রতি স্বামীর দ্বারা সহিংসতা/IPV সহায়তা চাওয়ার সাথে যুক্ত বাধাগুলো কমাতে কি করা যেতে পারে? আপনার মতামত জানাবেন।

**Key Informant Interview Guidelines: Male community members/ Male community leaders /Female community leaders**

**Socio-demographic Information:**

- Age
- Gender
- Educational status
- Occupation

**Information related to IPV:**

**What kind of IPVs are common in your community? Is there any particular reason behind the prevalence of particular type of IPV?**

- Physical
- Sexual
- emotional

**Individuals/Family / household-level influences:**

1. Do you think that age and family status of the male and female are related to the perpetration of IPV? If yes, how?
2. Do you think that men’s adverse childhood experiences are responsible for perpetrating IPV? If so, please explain.
3. Is there any connection between male’s and female’s education and perpetrating IPV? If so, please explain.
4. Is child marriage related to IPV? If so, please explain.
5. Do you think male’s unemployment is a reason of happening IPV?
6. Do you think man’s involvement in gambling or substance abuse or extramarital relationship/sex is related to IPV? Please explain.
7. Do you think that women’s open opportunity in income generating activities triggers violence against themselves?
8. As we are going through Covid-19 pandemic, do you think that Covid-19 has a connection with increased IPV? Can you please tell us about the factors that are responsible for IPV during Covid-19?

- Food availability, accessibility &affordability
- Employment status
- Financial aid (what/ whom)
- Anxiety due to uncertainty of being affected

**Community-level influences:**

1. Do you think widespread violence in the community encourages men to learn and practice violent behaviour? If so, please explain.
2. Are attitudes that condone violence and reinforce gender inequality is responsible for IPV? If so, please explain.
3. Do you think that negative attitudes towards divorced women is a reason for staying in an unhealthy or abusive relationship?

**Consequences of IPV:**

1. Can you please tell us about what types of consequences IPV have?

- Physical
- Psychological

1. Do you know that IPV has a significant impact on mental health? If so, please tell us how they are connected

**Current IPV support services**

1. Do you think women know about the current IPV support services and receive them when necessary?
2. What is your opinion regarding current IPV support services in Bangladesh?

- Level
- Quality
- Quantity: What services are available to IPV survivors seeking help? Are they available in all areas?

1. What are the challenges or faced by the victim of IPV in terms of receiving IPV support services?

- Social
- Cultural
- Economical
- Awareness and education related
- Stigma and Fear

**Recommendations:**

1. Do you have any suggestion on how IPV could be reduced? If so, Please tell us briefly.
2. What can be done to reduce the challenges associated with seeking IPV support?

**FGD Guideline: Mental Health Specialist/ Mental health Advocates/ Mental health Service provider**

**1. Socio demographic information**

- লিঙ্গ
- শিক্ষাগত যোগ্যতা
- অভিজ্ঞতা (বছর): আপনি কত বছর ধরে মানসিক স্বাস্থ্য সেবার সাথে জড়িত?
- এই মুহূর্তে আপনি কোথায় কাজ করছেন?
- বাংলাদেশে নারীর প্রতি সহিংসতার সামগ্রিক পরিস্থিতি সম্পর্কে আপনার মতামত কি?
- বর্তমান পরিস্থিতি
- সামাজিক এবং সাংস্কৃতিক অবস্থা
- আপনার মতে সাধারনত কি কি কারনে নারী সহিসতা ঘটতে পারে ।
- আপনার কাছে কোন বিষয়গুলোকে নারী সহিংসতার জন্য দায়ী মনে হয়?

**2. Individuals/Family / household-level influences:**

- - পুরুষ এবং মহিলাদের বয়স
  - পারিবারিক আয়
  - শিক্ষাগত যোগ্যতা
  - অল্প বয়সে বিয়ে বা বাল্যবিবাহ
  - কর্মসংস্থানের অবস্থা
  - জুয়া বা মাদক দ্রব্যের অপব্যবহার বা বিবাহ বহির্ভূত সম্পর্ক/যৌনতার অনুশীলন
  - কর্মসংস্থানে নারীদের অংশগ্রহণ

**3. Community-level influences:**

- আপনার মতে যে ধরনের মনভাব সহিংসতাকে মেনে নেয় এবং লিঙ্গবৈষম্যকে শক্তিশালী করে তোলে, তা কি নারী সহিসতার জন্য দায়ী? যদি হয়, আপনার নিজের ভাষায় ব্যাখ্যা করুন।
- আপনি কি মনে করেন যে সমাজের বিভিন্ন সহিংস ঘটনা পুরুষদের এই ধরনের আচরণ শিখতে এবং করতে উত্সাহিত করে? যদি তাই হয়, দয়া করে ব্যাখ্যা করুন।
- আপনি কি মনে হয় তালাকপ্রাপ্ত নারীদের প্রতি যে নেতিবাচক মনোভাব তা একটি অস্বাস্থ্যকর বা অমর্যাদাকর/ অত্যাচারিত/ অপমানজনক সম্পর্কের মধ্যে থাকার একটি কারণ?

**4. Intensity of the problems**

- কোভিড-১৯ মহামারী কীভাবে নিম্ন আয়ের জনসংখ্যার পারিবারিক পরিস্থিতিকে পরিবর্তন করেছে? এর মধ্যে প্রভাবশালী কারণগুলি কী কী?
- পারিবারিক স্ট্রেস/ গৃহস্থালির কাজের চাপ/ পারিবারিক চাপ
- শিশুদের শিক্ষা প্রতিষ্ঠান বন্ধ
- কোভিড-১৯ মহামারী কীভাবে নিম্ন আয়ের জনসংখ্যার সামাজিক এবং সাংস্কৃতিক অবস্থাকে পরিবর্তন করেছে? এর মধ্যে প্রভাবশালী কারণগুলি কী কী? (কোভিড-১৯ মহামারী কীভাবে বস্তির জনসংখ্যার সামাজিক অবস্থাকে পরিবর্তন করেছে?)
- চাকরি হারানো
- অর্থনৈতিক অনিশ্চয়তা
- চলাফেরায় বিধিনিষেধ প্রয়োগ
- আপনি কি মনে করেন কোভিড-১৯ মহামারীর স্বামীস্ত্রীর সম্পর্কের উপর কোনও প্রভাব রয়েছে? যদি হ্যাঁ হয়, তাহলে আপনি কেন এটি মনে করেন? আর যদি তা না হয়, তাহলে কেন নয়?

**5. Relationship of IPV and mental health**

- আপনি কি মনে করেন আইপিভি’র (সহিংসতার) মানসিক স্বাস্থ্যের উপর কোন প্রভাব আছে?
- আপনি কি কখনও এমন কোনও মহিলাকে (বিশেষত শহুরে বস্তির জনসংখ্যা বা বঞ্চিত/ প্রান্তিক জনসংখ্যার) খুঁজে পেয়েছেন যারা আইপিভির/সহিংসতার অভিজ্ঞতার পর তাদের মানসিক স্বাস্থ্য নিয়ে আপনার কাছে সহায়তা চেয়েছিলেন?
- আইপিভির অভিজ্ঞতা পরে নারীরা/ যারা সহংসিত হয়েছেন তারা কীভাবে মানসিক স্বাস্থ্য সহায়তা চান?

**6. Current mental health support for IPV survivors**

- বাংলাদেশে যারা সহংসিত হয়েছেন তাদের জন্য বর্তমান মানসিক স্বাস্থ্য সহায়তা সম্পর্কে আপনার মতামত কি?
- যারা সহংসিত হয়েছেন তারা কি ধরণের মানসিক স্বাস্থ্যসেবা পেতে পারেন? সহায়তা কি সব জায়গায় পাওয়া যায়?
- কোন কোন সেক্টর এর সাথে জড়িত (সংশ্লিষ্ট খাত)
- অন্যান্য কোন কোন সেক্টর/সংস্থার সাথে মিলিত/ একত্রিত
- আপনার কি মনে হয়, যারা সহংসিত হয়েছেন, তাদের জন্য বর্তমানে যে মানসিক স্বাস্থ্য সহায়তা রয়েছে, তা সম্পর্কে সবাই জানে?

**6. Barrier**

- যারা সহংসিত হয়েছেন তারা মানসিক স্বাস্থ্য সহায়তা পাওয়ার ক্ষেত্রে কী কী চ্যালেঞ্জের মুখোমুখি হন?
- সামাজিক
- সাংস্কৃতিক
- অর্থনৈতিক
- সচেতনতা এবং শিক্ষা সম্পর্কিত
- কলঙ্ক এবং ভয়

**7. Solution**

- আইপিভি সহায়তা/ সহিংসতা পরবর্তী সহায়তা চাওয়ার সাথে সম্পর্কিত চ্যালেঞ্জগুলি কমাতে কি কি করা যেতে পারে?
- আইপিভির শিকারদের/ যারা সহংসিত হয়েছেন তারা যেন মানসিক স্বাস্থ্য সহায়তা নিতে দেরি না করেন তার জন্য কি করনীয়?
- সমাজ থেকে সহিংসতা কমানোর উপায় কী?
- আপনার মতে সহিংসতা পরবর্তী সহায়তা উন্নত করতে প্রযুক্তি কি কি ভূমিকা পালন করতে পারে?
- ভালো অভ্যাস/ অনুশীলনের কি কোন উদাহরণ আছে? (তদন্ত: বিভিন্ন স্বাস্থ্য সেবা সংস্থার সাথে একত্রীকরণ, প্রাথমিক স্বাস্থ্যসেবার সাথে একত্রীকরণ, প্রযুক্তির ব্যবহার, বেসরকারী ও সরকারী খাতের সাথে কাজ করা)

**FGD guideline: Mental Health Specialist/ Mental health Advocates/ Mental health Service providers (English)**

**1. Socio demographic information**

- Gender
- Educational qualification
- Years of experience: For how long have you been in mental health service provision?
- place of practicing

**What is your perspective about overall situation of Intimate partner violence in Bangladesh?**

- Current situation
- Socio- cultural aspect
- What were the common context of IPV?

**What are the drivers of IPV in your perspective?**

**2. Individuals/Family / household-level influences:**

- Age of the male and female
- Household income
- Education level.
- Early marriage or childhood marriage
- Employment status
- Practice of gambling or substance abuse or extramarital relationship/sex
- Women’s participation in employment

**3. Community-level influences:**

1. Are attitudes that condone violence and reinforce gender inequality is responsible for IPV? If so, please explain.
2. Do you think that negative attitudes towards divorced women is a reason for staying in an unhealthy or abusive relationship?

**4. Intensity of the problems**

- How COVID-19 pandemic has changed the household scenario of low-income population? What are the influential factors?
  - Household stress
  - Closure of educational institution of children
- How COVID-19 pandemic has changed the social cultural scenario of low-income population? What are the influential factors?
  - Job loss
  - Economic uncertainty
  - Mobility restriction
- Do you think COVID-19 pandemic has any influence on intimate relationship? If yes, then why do you think that? And if not then why?

**5. Relationship of IPV and mental health**

- Do you think IPV has any effect on mental health?
- Have you ever found any women (specially from urban slum population or marginalized population) who experienced IPV sought mental health support from you?
- How do women seek mental health support after experiencing IPV?

**6. Current mental health support for IPV survivors**

- What is your opinion regarding current mental health support for IPV survivors in Bangladesh?
- What kind of mental health services are available to IPV survivors seeking help? Are they available in all areas?
- Sector involved
- Integration with other services
- Do you think people know about the current mental health services for supporting IPV survivors?

1. **Barrier**

- What are the challenges or faced by the victim of IPV in terms of receiving mental health support services?
  - - Social
    - Cultural
    - Economical
    - Awareness and education related
    - Stigma and Fear

1. **Solution**

- What can be done to reduce the challenges associated with seeking IPV support?
- How to reduce the delays seeking mental health support among the victim of IPV?
- What are the solutions to reduce IPV from the society?
- What do you think about the role that technology can play in help improve access to IPV support?
- Are there any examples of good practice? (probe: integration within different health services, integrating in the primary care, use of technology, working with private and public sector)

**Key Informant Interview Guideline: Gender specialist/ Gender advocates**

1. Socio demographic information
   - লিঙ্গ
   - শিক্ষাগত যোগ্যতা
   - অভিজ্ঞতা (বছর): আপনি কত বছর ধরে মানসিক স্বাস্থ্য সেবার সাথে জড়িত? এই মুহূর্তে আপনি কোথায় কাজ করছেন?

- আপনার কাছে কোন বিষয়গুলোকে নারী সহিংসতার জন্য দায়ী মনে হয়?

1. Individuals/Family / household-level influences:
   - পুরুষ এবং মহিলাদের বয়স
   - পারিবারিক আয়
   - শিক্ষাগত যোগ্যতা
   - অল্প বয়সে বিয়ে বা বাল্যবিবাহ
   - কর্মসংস্থানের অবস্থা
   - জুয়া বা মাদক দ্রব্যের অপব্যবহার বা বিবাহ বহির্ভূত সম্পর্ক/যৌনতার অনুশীলন
   - কর্মসংস্থানে নারীদের অংশগ্রহণ
2. Community-level influences:

- আপনার মতে যে ধরনের মনভাব সহিংসতাকে মেনে নেয় এবং লিঙ্গবৈষম্যকে শক্তিশালী করে তোলে, তা কি নারী সহিসতার জন্য দায়ী? যদি হয়, আপনার নিজের ভাষায় ব্যাখ্যা করুন।
- আপনি কি মনে হয় তালাকপ্রাপ্ত নারীদের প্রতি যে নেতিবাচক মনোভাব তা একটি অস্বাস্থ্যকর বা অমর্যাদাকর/ অত্যাচারিত/ অপমানজনক সম্পর্কের মধ্যে থাকার একটি কারণ?

4. Intensity of the problems

- কোভিড-১৯ মহামারী কীভাবে নিম্ন আয়ের জনসংখ্যার পারিবারিক পরিস্থিতিকে পরিবর্তন করেছে? এর কারণগুলি কী কী?
- পারিবারিক স্ট্রেস/ গৃহস্থালির কাজের চাপ/ পারিবারিক চাপ
- শিশুদের শিক্ষা প্রতিষ্ঠান বন্ধ হওয়া
- কোভিড-১৯ মহামারী কীভাবে নিম্ন আয়ের জনসংখ্যার সামাজিক এবং সাংস্কৃতিক অবস্থাকে পরিবর্তন করেছে? এর কারণগুলি কী কী? (কোভিড-১৯ মহামারী কীভাবে নিম্ম আয়ের জনসংখ্যার সামাজিক অবস্থাকে পরিবর্তন করেছে?)
- চাকরি হারানো
- অর্থনৈতিক অনিশ্চয়তা
- চলাফেরায় বিধিনিষেধ
- আপনি কি মনে করেন কোভিড-১৯ মহামারীর স্বামী-স্ত্রীর সম্পর্কের উপর কোনও প্রভাব রয়েছে? যদি হ্যাঁ হয়, তাহলে আপনি কেন এটি মনে করেন? আর যদি তা না হয়, তাহলে কেন নয়?

5. Relationship of IPV and mental health

- আপনি কি মনে করেন আইপিভি’র (সহিংসতার) মানসিক স্বাস্থ্যের উপর কোন প্রভাব আছে?
- আপনি কি কখনও এমন কোনও মহিলাকে (বিশেষত শহুরে নিম্ম আয়ের বা সুবিধা বঞ্চিত/ প্রান্তিক জনসংখ্যার) খুঁজে পেয়েছেন যারা আইপিভির/সহিংসতার অভিজ্ঞতার পর তাদের মানসিক স্বাস্থ্য নিয়ে আপনার কাছে সহায়তা চেয়েছিলেন?
- আইপিভির অভিজ্ঞতা পরে নারীরা/ যারা সহংসিত হয়েছেন তারা কীভাবে মানসিক স্বাস্থ্য সহায়তা চান?

6. Current mental health support for IPV survivors

- আইপিভি সারভাইভারদের জন্য বর্তমান মানসিক স্বাস্থ্য সহায়তা/ সহিংসতা অভিজ্ঞতাপ্রাপ্ত নারীদের জন্য বর্তমান মানসিক স্বাস্থ্য সহায়তা
- বাংলাদেশে যারা সহংসিত হয়েছেন তাদের জন্য বর্তমান মানসিক স্বাস্থ্য সহায়তা সম্পর্কে আপনার মতামত কি?
- যারা সহংসিত হয়েছেন তারা কি ধরণের মানসিক স্বাস্থ্যসেবা পেতে পারেন? সহায়তা কি সব জায়গায় পাওয়া যায়?
- কোন কোন সেক্টর এর সাথে জড়িত (সংশ্লিষ্ট খাত)
- অন্যান্য কোন কোন সেক্টর/সংস্থার সাথে মিলিত/ একত্রিত
- আপনার কি মনে হয়, যারা সহংসিত হয়েছেন, তাদের জন্য বর্তমানে যে মানসিক স্বাস্থ্য সহায়তা রয়েছে, তা সম্পর্কে সবাই জানে?

7. Barrier:

- যারা সহংসিত হয়েছেন তারা মানসিক স্বাস্থ্য সহায়তা পাওয়ার ক্ষেত্রে কী কী চ্যালেঞ্জের মুখোমুখি হন?
- সামাজিক
- সাংস্কৃতিক
- অর্থনৈতিক
- সচেতনতা এবং শিক্ষা সম্পর্কিত
- কলঙ্ক এবং ভয়

8. Solutions:

- আইপিভি সহায়তা/ সহিংসতা পরবর্তী সহায়তা চাওয়ার সাথে সম্পর্কিত চ্যালেঞ্জগুলি কমাতে কি কি করা যেতে পারে?
- আইপিভির শিকারদের/ যারা সহংসিত হয়েছেন তারা যেন মানসিক স্বাস্থ্য সহায়তা নিতে দেরি না করেন তার জন্য কি করনীয়?
- সমাজ থেকে সহিংসতা কমানোর উপায় কী?
- আপনার মতে সহিংসতা পরবর্তী সহায়তা উন্নত করতে প্রযুক্তি কি কি ভূমিকা পালন করতে পারে?
- ভালো অভ্যাস/ অনুশীলনের কি কোন উদাহরণ আছে? (তদন্ত: বিভিন্ন স্বাস্থ্য সেবা সংস্থার সাথে একত্রীকরণ, প্রাথমিক স্বাস্থ্যসেবার সাথে একত্রীকরণ, প্রযুক্তির ব্যবহার, বেসরকারী ও সরকারী খাতের সাথে কাজ করা)

**Key Informant Interview Guideline: Gender specialist/ Gender advocates**

1. **Socio demographic information**

- Age
- Gender
- Educational qualification
- Years of experience:
- Year and place of practicing

1. **What is your perspective about overall situation of Intimate partner violence in Bangladesh?**

- Current situation
- Socio- cultural aspect
- What were the common context of IPV?

1. **What are the drivers of IPV in your perspective?**
2. **Individuals/Family / household-level influences:**

- Age of the male and female
- Household income
- Education level.
- Early marriage or childhood marriage
- Employment status
- Practice of gambling or substance abuse or extramarital relationship/sex
- Women’s participation in employment

1. **Community-level influences:**
2. Are attitudes that condone violence and reinforce gender inequality is responsible for IPV? If so, please explain.
3. Do you think that negative attitudes towards divorced women is a reason for staying in an unhealthy or abusive relationship?

- **Intensity of the problems**

1. How COVID-19 pandemic has changed the household scenario of low-income population? What are the influential factors?

- Household stress
- Closure of educational institution of children

1. How COVID-19 pandemic has changed the social scenario of slum population? What are the influential factors?
2. Do you think COVID-19 pandemic has any influence on intimate relationship? If yes, then why do you think that? And if not then why?

- **Relationship of IPV and mental health**

1. Do you think IPV has any effect on mental health?
2. Have you ever supported any women (specially from urban slum population or marginalized population) who experienced IPV to seek mental health support?
3. How do women seek mental health support after experiencing IPV?

- **Current mental health support for IPV survivors**

1. What is your opinion regarding IPV support for IPV survivors in Bangladesh?
2. What is your opinion regarding current mental health support for IPV survivors in Bangladesh?
   1. What kind of mental health services are available to IPV survivors seeking help? Are they available in all areas?
   2. Sector involved
   3. Integration with other services
3. **Barrier**
4. What are the challenges or faced by the victim of IPV in terms of receiving IPV support services?
5. What are the challenges or faced by the victim of IPV in terms of receiving mental health support services?
   - - Social
     - Cultural
     - Economical
     - Awareness and education related
     - Stigma and Fear
6. **Solution**

- What can be done to reduce the challenges associated with seeking IPV support?
- How to reduce the delays seeking mental health support among the victim of IPV?
- What are the solutions to reduce IPV from the society?
- What do you think about the role that technology can play in help improve access to IPV support?

1. Are there any examples of good practice? (probe: integration within different health services, integrating in the primary care, use of technology, working with private and public sector
