# Supplementary material for: Exploring the pattern of mental health support-seeking behaviour and related barriers among women experiencing intimate partner violence in urban slums of Bangladesh: perspectives from multiple level stakeholders
Source: PLOS Glob Public Health. 2025 May 9;5(5):e0004568. doi: 10.1371/journal.pgph.0004568 (PMC12063865; doi:10.1371/journal.pgph.0004568)
Supplement: S1 Table — (DOCX) [file pgph.0004568.s002.docx]

| **S1 Table: Identifications of Stakeholders** | | |  |  |
| --- | --- | --- | --- | --- |
| **Type of stakeholders** | **Identification** | **Years of involvement in current position** |  |  |
| 1. Gender specialist | GST-KII-01 | 15 years | |  |
|  | GST-KII-02 | 8 years | |  |
|  | GST-KII-03 | 12 years | |  |
|  | GST-KII-04 | 10 years | |  |
|  | GST-KII-05 | 12 years | |  |
|  | GST-KII-06 | 6 years | |  |
|  | GST-KII-07 | 7 years | |  |
| 2. Healthcare Provider | HCP-KII-01 | 6 years | |  |
|  | HCP-KII-02 | 25 years | |  |
|  | HCP-KII-03 | 14 years | |  |
|  | HCP-KII-04 | 10 Year | |  |
|  | HCP-KII-05 | 2 years | |  |
|  | HCP-KII-06 | 7 years | |  |
| 3. Mental healthcare providers | MHP-FGD-01 | 10 years | |  |
|  | MHP-FGD-02 | 5 years | |  |
|  | MHP-FGD-03 | 10 years | |  |
|  | MHP-FGD-04 | 7 years | |  |
|  | MHP-FGD-05 | 7 years | |  |
|  | MHP-FGD-06 | 9 years | |  |
|  | MHP-FGD-07 | 6 years | |  |
|  | MHP-FGD-08 | 7 years | |  |

| **Type of stakeholders** | **Identification** | **Age in years** |  |
| --- | --- | --- | --- |
| **Community Leader (Male)** | ML-KII-01 | 65 years |  |
|  | ML-KII-02 | 42 years |  |
|  | ML-KII-03 | 35 years |  |
|  | ML-KII-04 | 30 years |  |
|  | ML-KII-05 | 35 years |  |
|  | ML-KII-06 | 40 years |  |
|  | ML-KII-07 | 42 years |  |
| **Community Leader (Female)** | FL-KII-01 | 45 years |  |
|  | FL-KII-02 | 55 years |  |
|  | FL-KII-03 | 40 years |  |
|  | FL-KII-04 | 55 years |  |
|  | FL-KII-05 | 35 years |  |
|  | FL-KII-06 | 65 years |  |
|  | FL-KII-07 | 56 years |  |
| **Male slum dweller** | CM-IDI-01 | 38 years |  |
|  | CM-IDI-02 | 42 years |  |
|  | CM-IDI-03 | 30 years |  |
|  | CM-IDI-04 | 35 years |  |
|  | CM-IDI-05 | 37 years |  |
|  | CM-IDI-06 | 45 years |  |
|  | CM-IDI-07 | 42 years |  |
|  | CM-IDI-08 | 54 years |  |
|  | CM- IDI-09 | 34 years |  |
|  | CM-IDI-10 | 35 years |  |
|  | CM-IDI-11 | 32 years |  |
| **Female slum dweller** | CF-IDI-01 | 23 years |  |
|  | CF-IDI-02 | 22 years |  |
|  | CF-IDI-03 | 38 years |  |
|  | CF-IDI-04 | 30 years |  |
|  | CF-IDI-05 | 60 years |  |
|  | CF-IDI-06 | 34 years |  |
|  | CF-IDI-07 | 30 years |  |
|  | CF-IDI-08 | 30 years |  |
|  | CF-IDI-09 | 22 years |  |
|  | CF-IDI-10 | 43 years |  |
|  | CF-IDI-11 | 40 years |  |
|  | CF-IDI-12 | 42 years |  |
|  | CF-IDI-13 | 45 years |  |
