# Supplementary material for: Exploring the pattern of mental health support-seeking behaviour and related barriers among women experiencing intimate partner violence in urban slums of Bangladesh: perspectives from multiple level stakeholders
Source: PLOS Glob Public Health. 2025 May 9;5(5):e0004568. doi: 10.1371/journal.pgph.0004568 (PMC12063865; doi:10.1371/journal.pgph.0004568)
Supplement: S1 Checklist — (DOCX) [file pgph.0004568.s003.docx]

**S1 Checklist: COREQ (Consolidated criteria for Reporting Qualitative research) Checklist**

| Topic | Item No. | Guide Questions/Description | Remark | Reported on Page No. |
| --- | --- | --- | --- | --- |
| Domain 1: Research team and reflexivity | | | | |
| Personal characteristics | | | | |
| Interviewer/facilitator | 1 | Which author/s conducted the interview or focus group? | Lead author and four research team members | Page 6, Method |
| Credentials | 2 | What were the researcher’s credentials? E.g. PhD, MD | Mentioned in the section Author credentials | Page 30, Authors credential |
| Occupation | 3 | What was their occupation at the time of the study? | Mentioned in the section Author credentials | Page 30, Authors credential |
| Gender | 4 | Was the researcher male or female? | Female: 6 | N/A |
| Experience and training | 5 | What experience or training did the researcher have? | Lead author and other co-authors are experienced in conducting both qualitative research designs. | Page 6, Method |
| Relationship with participants | | | | |
| Relationship established | 6 | Was a relationship established prior to study commencement? | No relationship with the participants was established before the commencement of the study. | N/A |
| Participant knowledge of the interviewer | 7 | What did the participants know about the researcher? e.g. personal goals, reasons for doing the research | The lead author introduced herself to participants stating she is a medical professional and a global mental health researcher, etc. as well as describing the research team, the purpose of the project and answering any questions participants may have had about the study and those involved in it. | N/A |
| Interviewer characteristics | 8 | What characteristics were reported about the inter viewer/facilitator? e.g. Bias, assumptions, reasons and interests  in the research topic | The interviewer was a medical professional with a MSc degree in Global Mental Health, involved in multiple mental health research in Bangladesh and also had read the literature on mental health care pathways prior to beginning the study. | Page 30, Authors credential |
| Domain 2: Study design | | | | |
| Theoretical framework | | | | |
| Methodological orientation and Theory | 9 | What methodological orientation was stated to underpin the study? e.g.  grounded theory, discourse analysis, ethnography, phenomenology, content analysis | Methodological orientation:  Exploratory, descriptive qualitative research methodology. Thematic analyses were applied for data analysis using both inductive and deductive techniques. | Page 4, Method |
| Participant selection | | | | |
| Sampling | 10 | How were participants selected? e.g. purposive, convenience, consecutive, snowball | The study used both purposive and snowball sampling as the combination of these techniques helps identify information from participants who are experienced or knowledgeable about the subject matter. We identified potential participants from slum communities by the icddrb UHDSS team members who have been working there for seven years. Authors also used existing collaborations with the research team for identifying potential participants | Page 6, Method |
| Method of approach | 11 | How were participants approached? e.g. face-to-face, telephone, mail, email | Both data collection techniques – face-to-face and online – were frequently used in qualitative studies. | Page 6, Method |
| Sample size | 12 | How many participants were in the study? | **Total Number of participants**= 59  **In-depth Interview (IDI)**  Female slum dweller (n=13)  Male slum dweller (n=11)  **Key informant interviews (KII)**  Male Community leaders (n=7)  Female community leaders (n=7)  Gender specialists (n=8)  Health service providers (n=6)  **Focus group discussions**  Mental health service providers (psychologists) (n=7) | Page 7, Method |
| Non-participation | 13 | How many people refused to participate or dropped out? Reasons? | No one | N/A |
| Setting | | | | |
| Setting of data collection | 14 | Where was the data collected? e.g. home, clinic, workplace | We conducted our study in five selected slums covered by the Urban Health and Demographic Surveillance Systems (UHDSS) of International Centre for Diarrhoeal Disease Research, Bangladesh (icddr, b), namely Korail, Mirpur, Shampur, Dholpur, and Tongi-Ershadnagar in Dhaka (North & South) and Gazipur City Corporation. We also collected data using online meeting platforms | Page 6., Method |
| Presence of nonparticipants | 15 | Was anyone else present besides the participants and researchers? | none | N/A |
| Description of sample | 16 | What are the important characteristics of the sample? e.g. demographic data, date | Reported in the result section | Page 8 |
| Data collection | | | | |
| Interview guide | 17 | Were questions, prompts, guides provided by the authors? Was it pilot tested? | Yes interview guides were pilot tested. All questions are provided in supplement 2. | N/A |
| Repeat interviews | 18 | Were repeat interviews carried out? If yes, how many? | There was no repeat interview. | N/A |
| Audio/visual recording | 19 | Did the research use audio or visual recording to collect the data? | All interviews were audio-recorded after taking consent from the participants and were transcribed by trained research team members. | Page 7, Method |
| Field notes | 20 | Were field notes made during and/or after the interview or focus group? | Yes, notes were taken by the research team members | Page 6, Method |
| Duration | 21 | What was the duration of the interviews or focus group? | The average time of interviews was 30-40 minutes. | Page 6, Method |
| Data saturation | 22 | Was data saturation discussed? | Data saturation reached when no new data emerged from the interviews. | N/A |
| Transcripts returned | 23 | Were transcripts returned to participants for comment and/or correction? | No | N/A |
| Domain 3: analysis and findings | | | | |
| Data analysis | | | | |
| Number of data coders | 24 | How many data coders coded the data? | 5 | Page 6-7, Method |
| Description of the coding tree | 25 | Did authors provide a description of the coding tree? | Yes | Figure 1 |
| Derivation of themes | 26 | Were themes identified in advance or derived from the data? | Derived from the data | Page 7 |
| Software | 27 | What software, if applicable, was used to manage the data? | No | Page 7 |
| Participant checking | 28 | Did participants provide feedback on the findings? | No | N/A |
| Reporting | | | | |
| Quotations presented | 29 | Were participant quotations presented to illustrate the themes/findings?  Was each quotation identified? e.g. participant number | Yes | Page 8-25, Result |
| Data and findings consistent | 30 | Was there consistency between the data presented and the findings? | Yes | Page 8-25, Result |
| Clarity of major themes | 31 | Were major themes clearly presented in the findings? | Yes | Page 8-25, Result |
| Clarity of minor themes | 32 | Is there a description of diverse cases or discussion of minor themes? | Yes | Page 8-25, Result |
